# Supplementary material for: Mitochondrial subpopulations in oocytes and cumulus cells exhibit distinct age-associated changes and selective plasticity in response to NMN supplementation
Source: J Ovarian Res. 2026 Aug 7;19:332. doi: 10.1186/s13048-026-02223-6 (PMC13732867; doi:10.1186/s13048-026-02223-6)

Aged Cumulus Cells

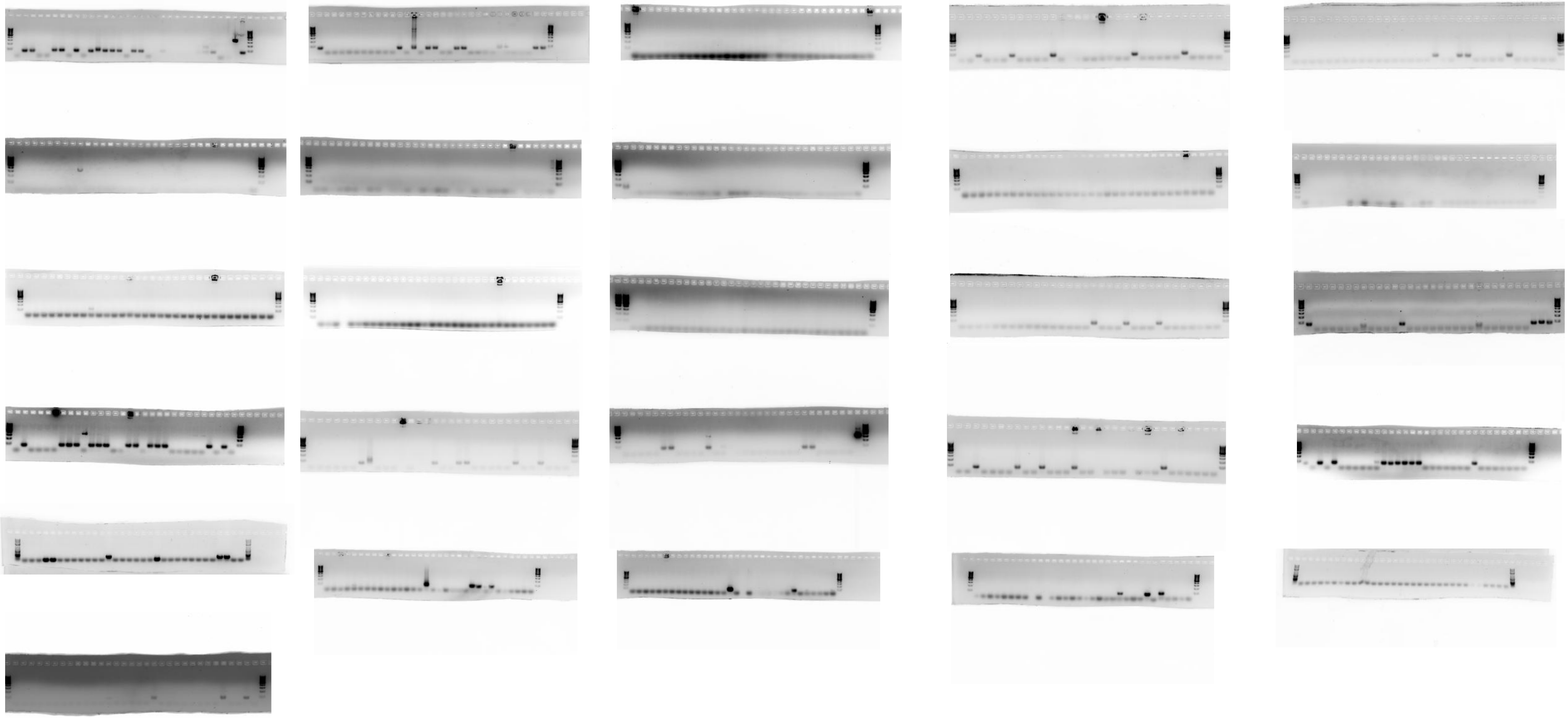

Aged Cumulus Cells

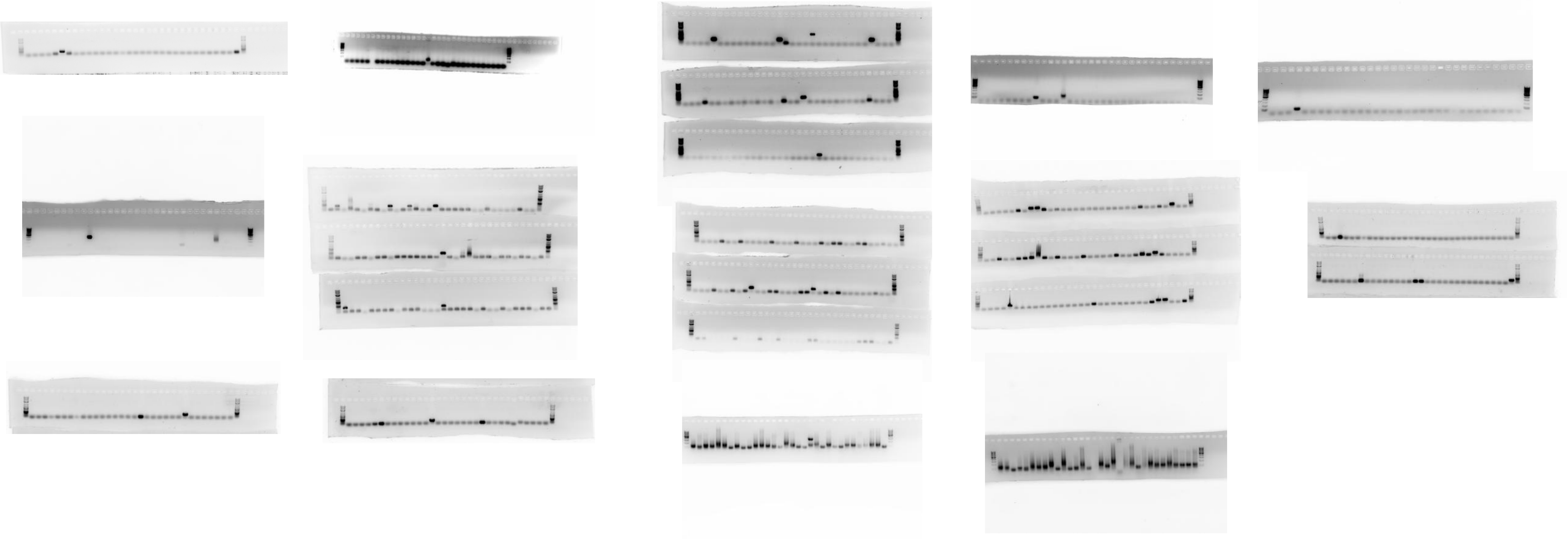

Small Aged Cumulus Cells

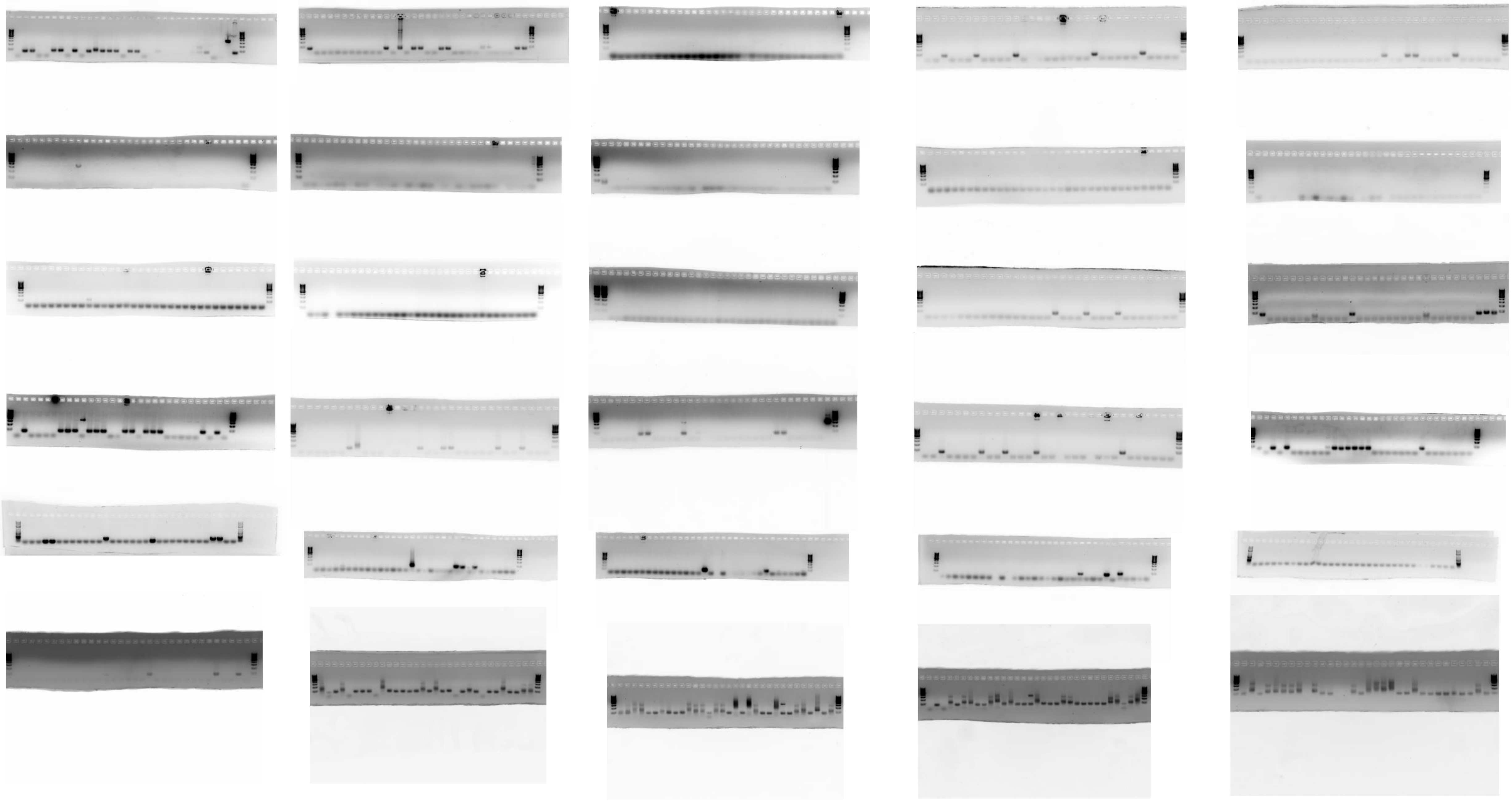

Small Aged Cumulus Cells

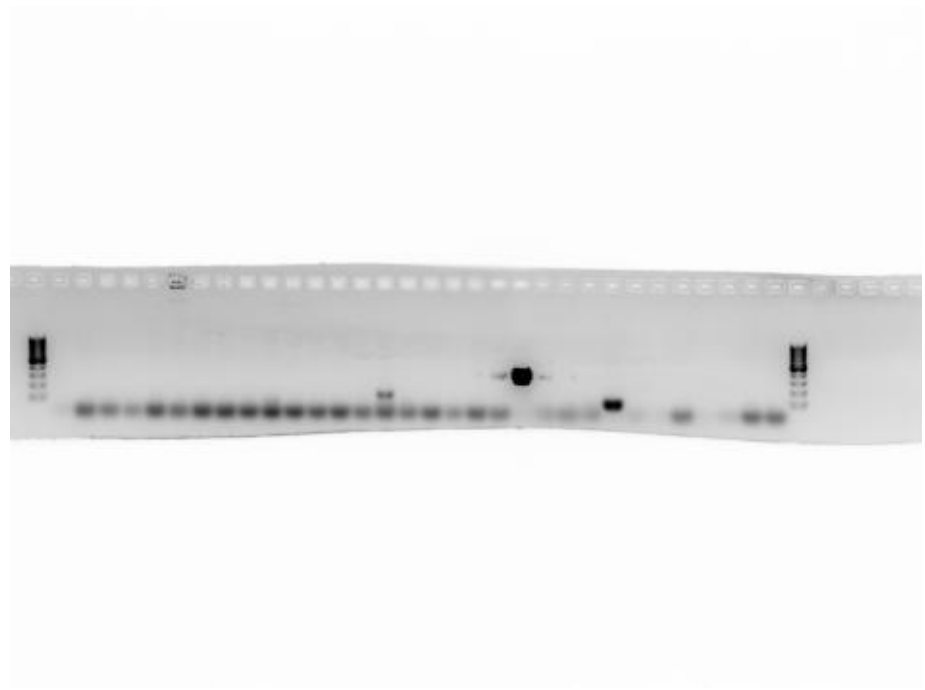

Large Aged Cumulus Cells

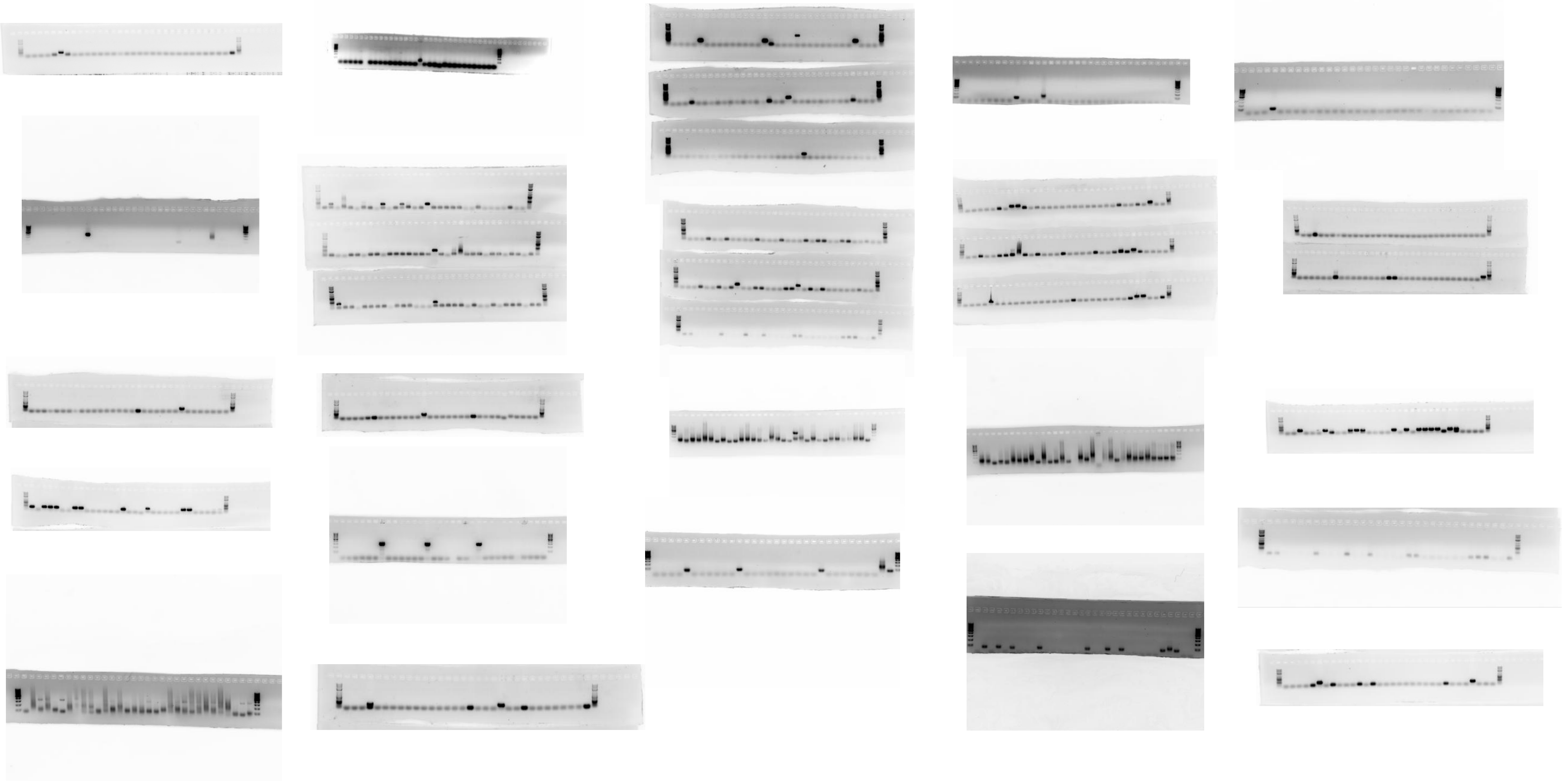

Large Aged Cumulus Cells

Low ΔΨm Aged Cumulus Cells

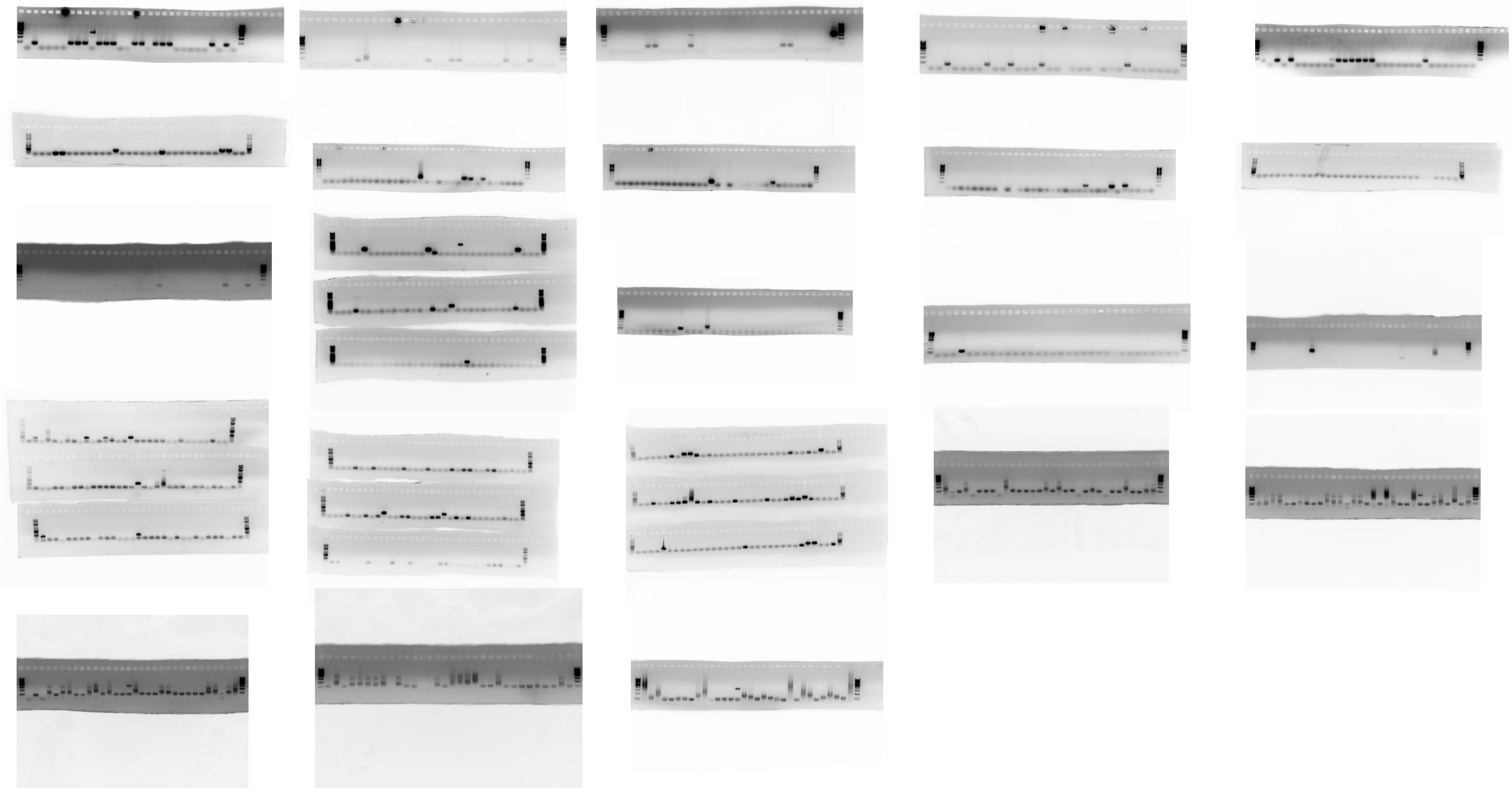

High ΔΨm Aged Cumulus Cells

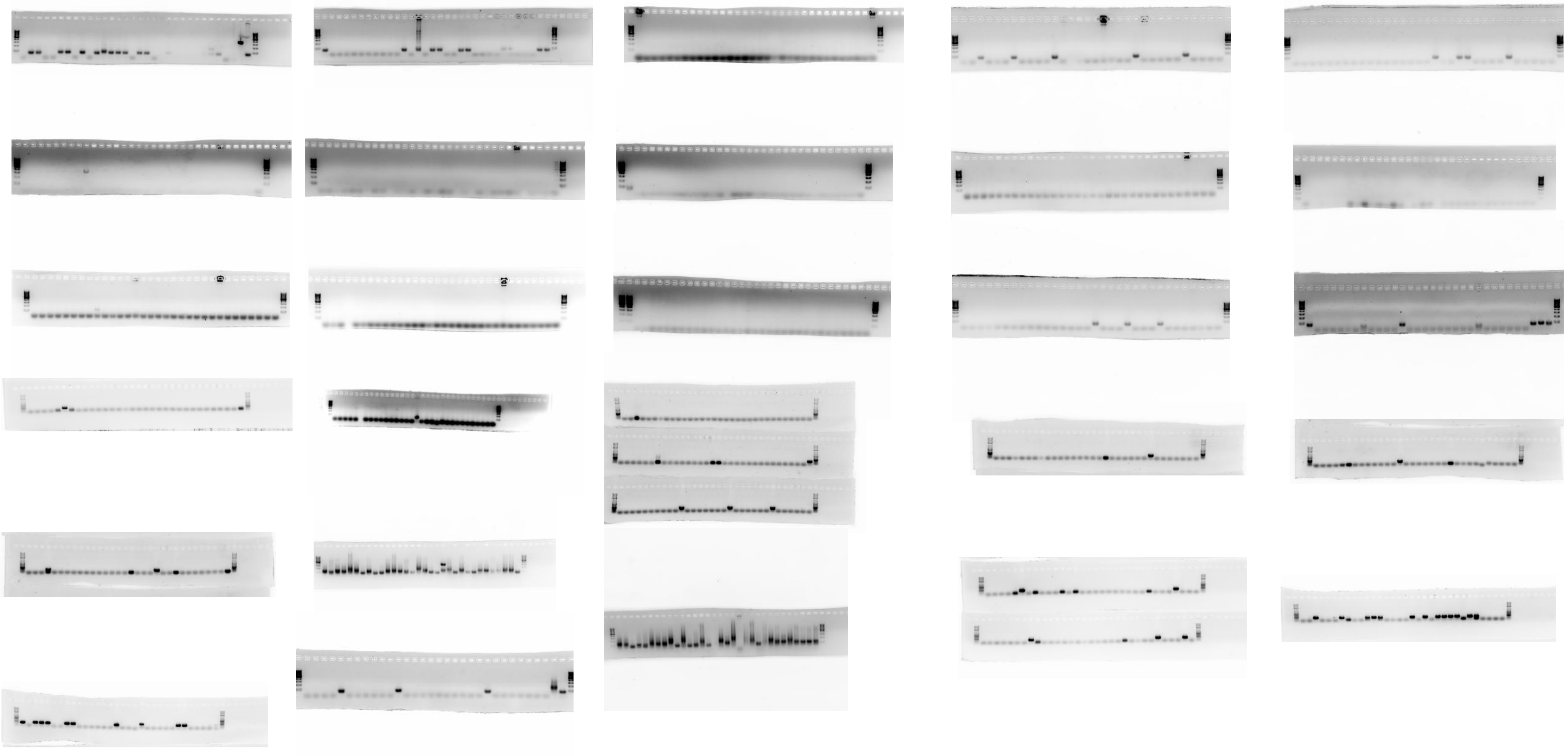

High ΔΨm Aged Cumulus Cells

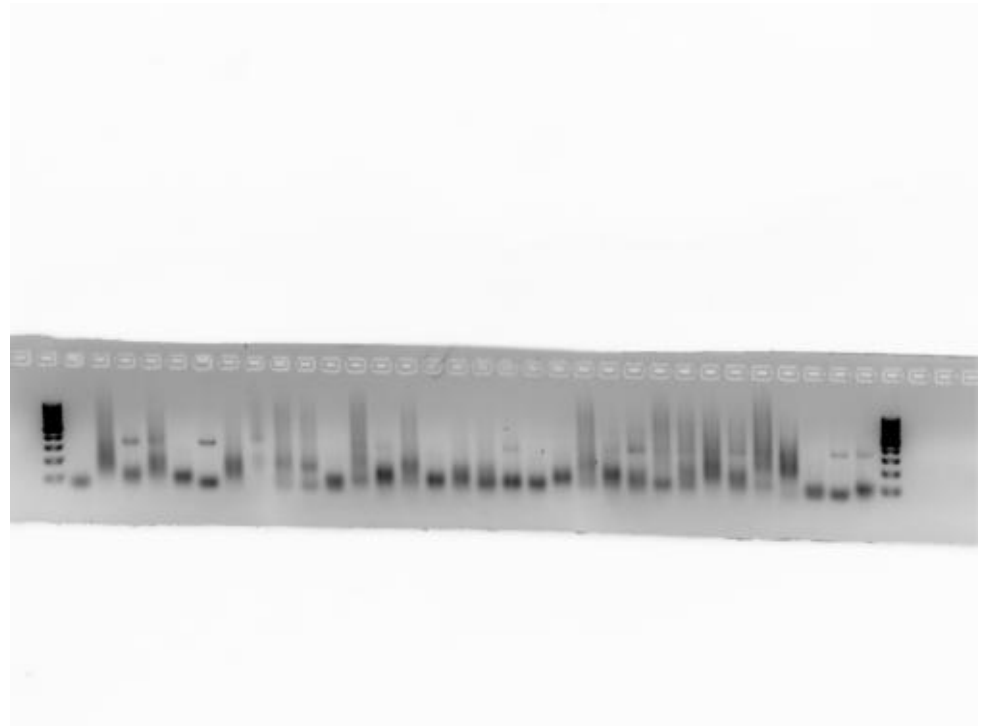

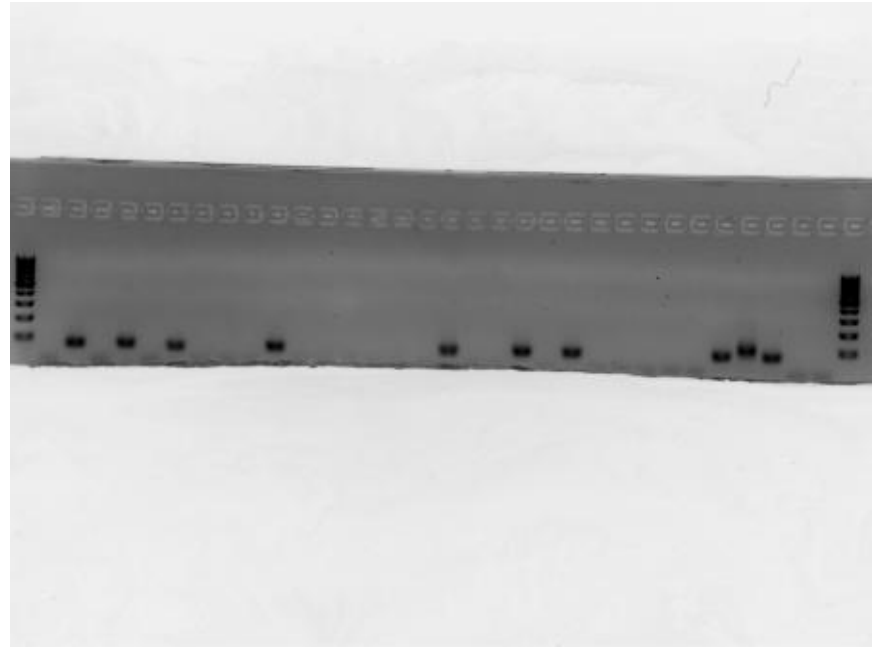

Supplement: Supplementary file 1 [file 13048_2026_2223_MOESM1_ESM.pdf]
