# Supplementary material for: Mitochondrial subpopulations in oocytes and cumulus cells exhibit distinct age-associated changes and selective plasticity in response to NMN supplementation
Source: J Ovarian Res. 2026 Aug 7;19:332. doi: 10.1186/s13048-026-02223-6 (PMC13732867; doi:10.1186/s13048-026-02223-6)

Aged Oocytes

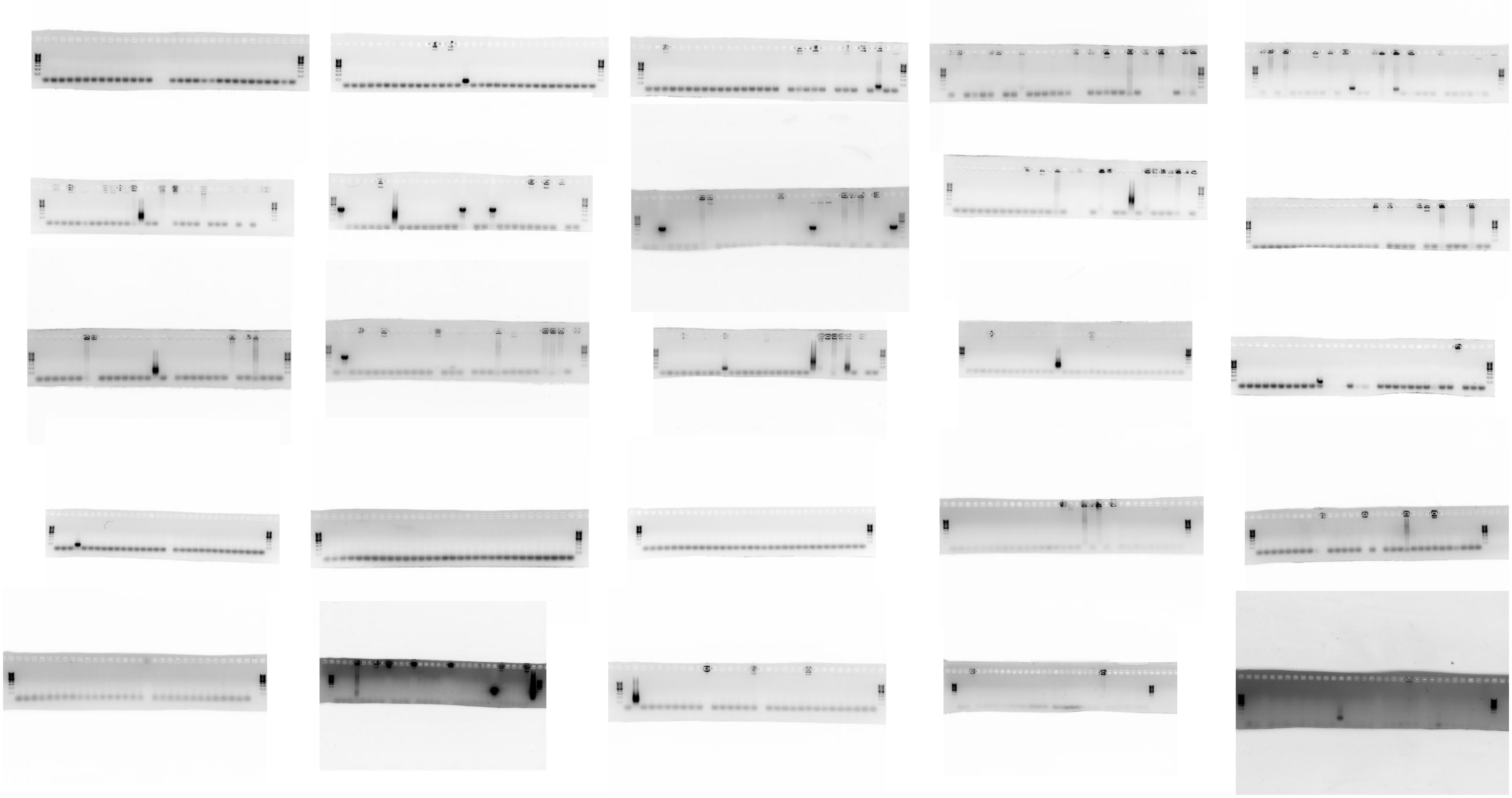

Aged Oocytes

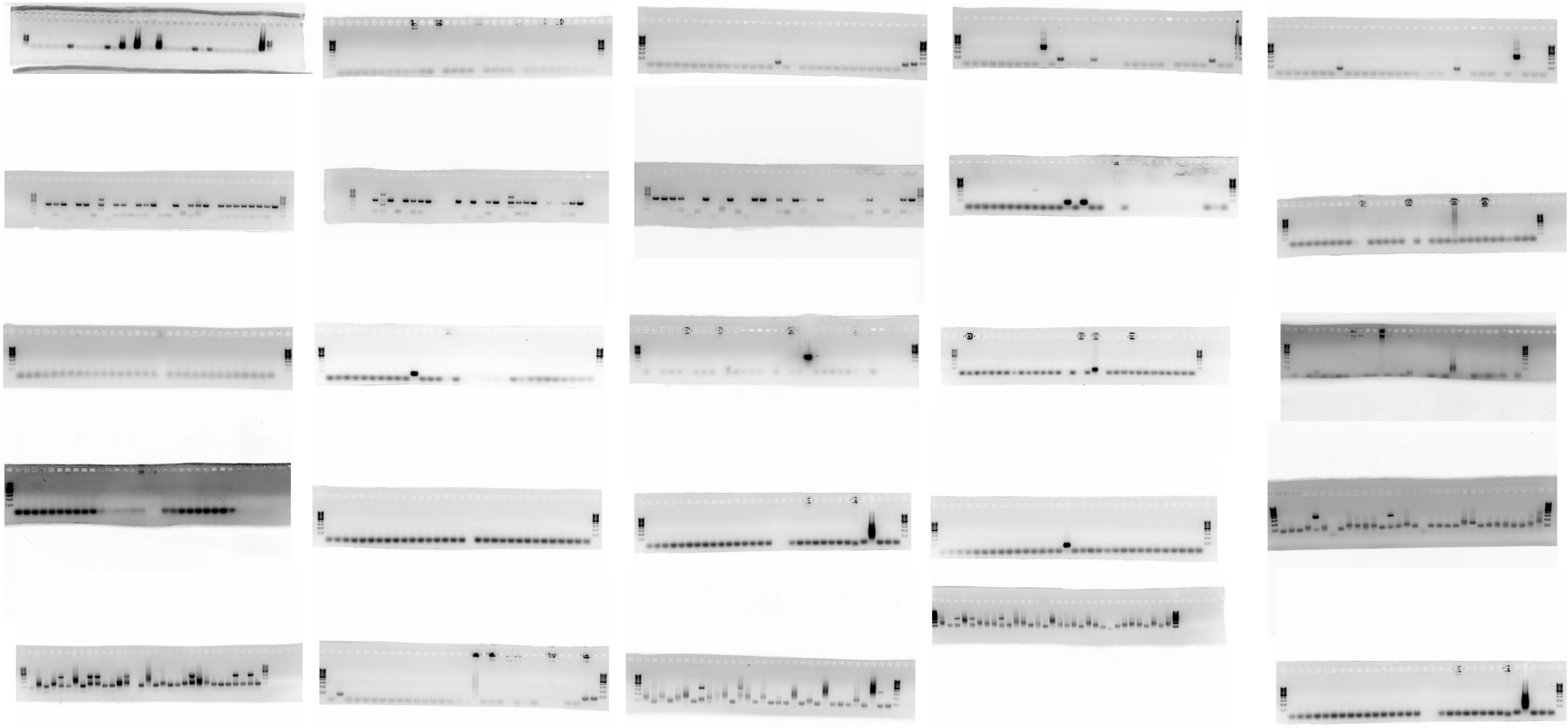

Aged Oocytes

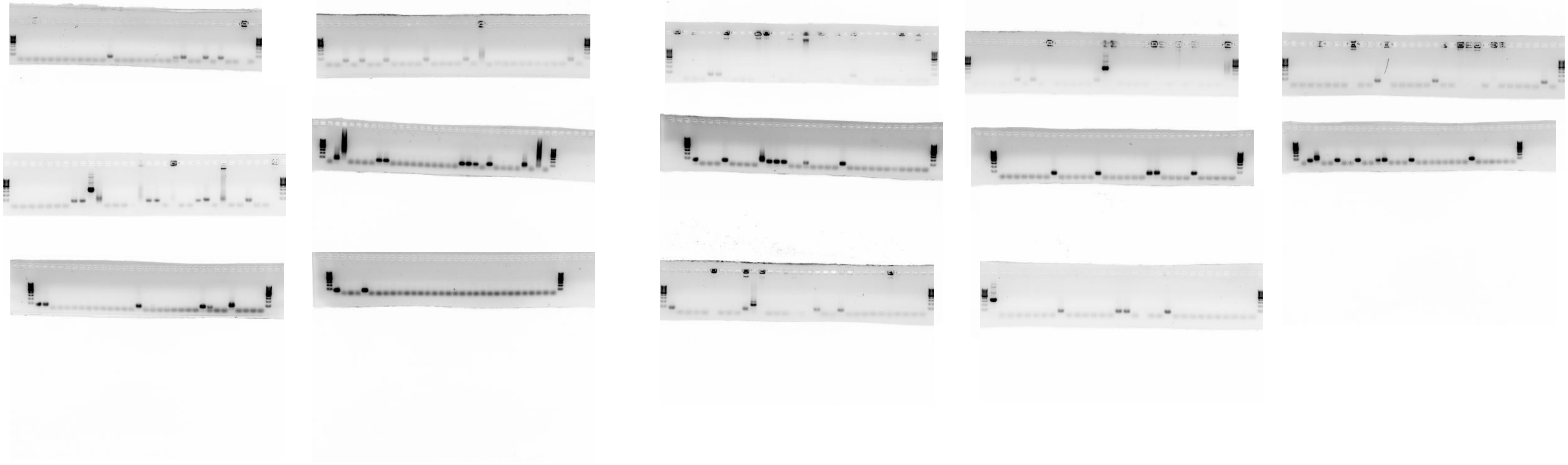

Small Aged Oocytes

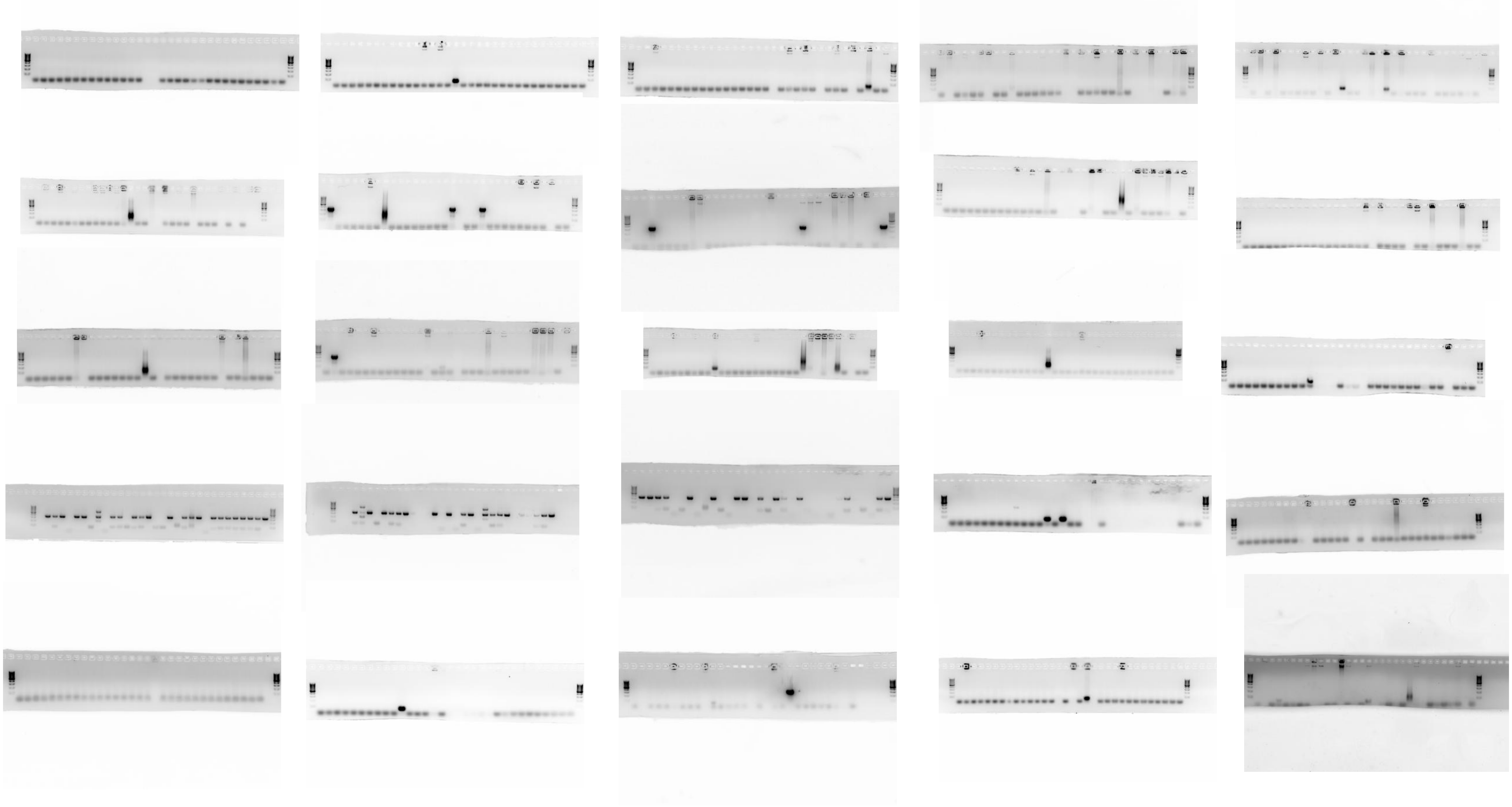

Small Aged Oocytes

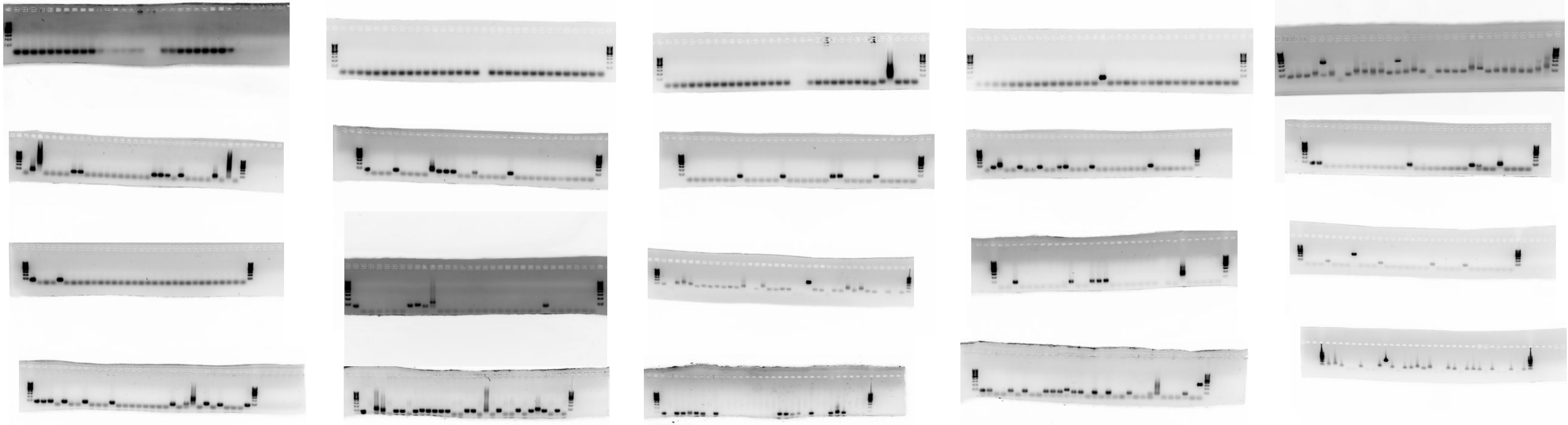

Large Aged Oocytes

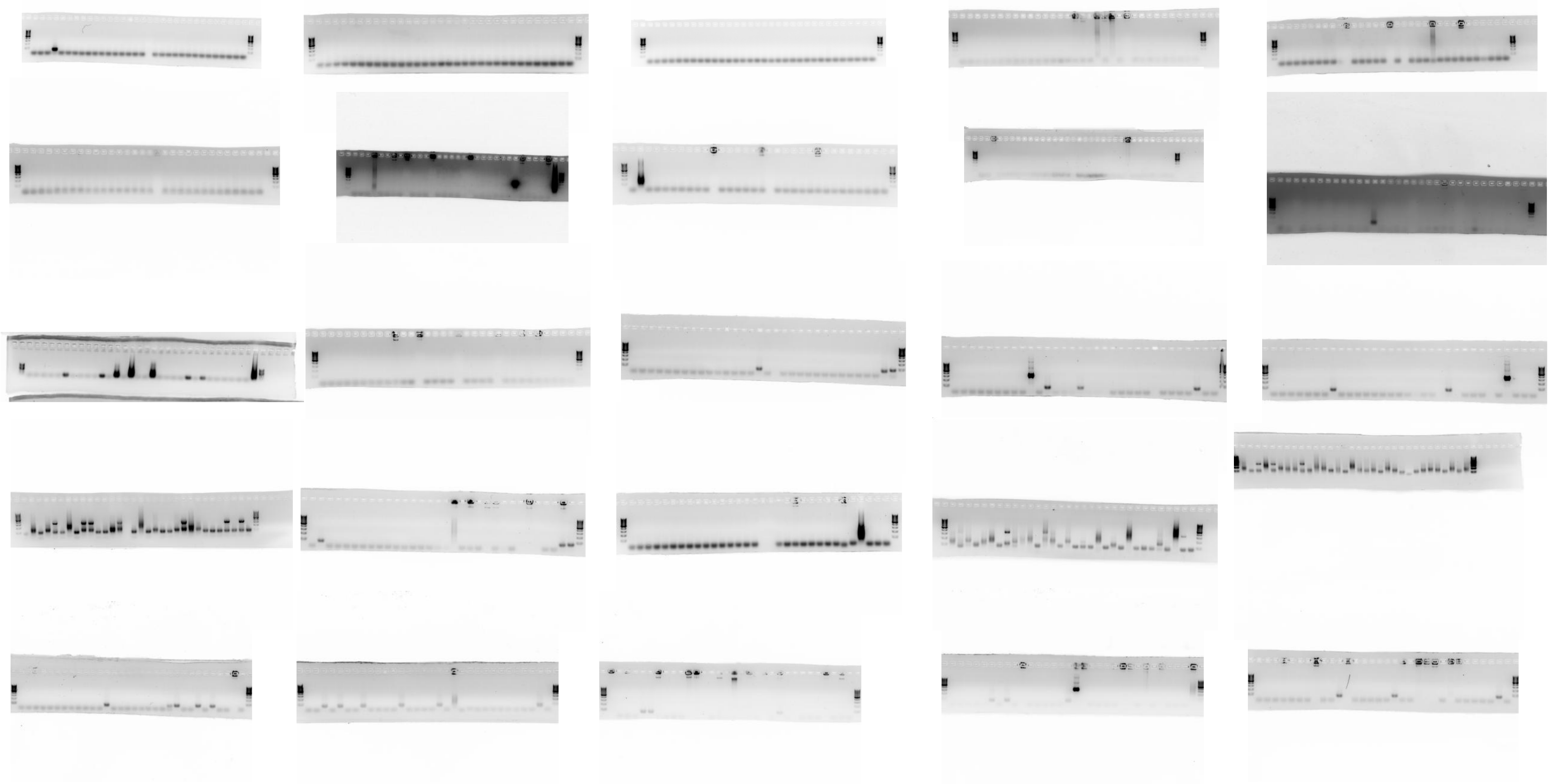

Large Aged Oocytes

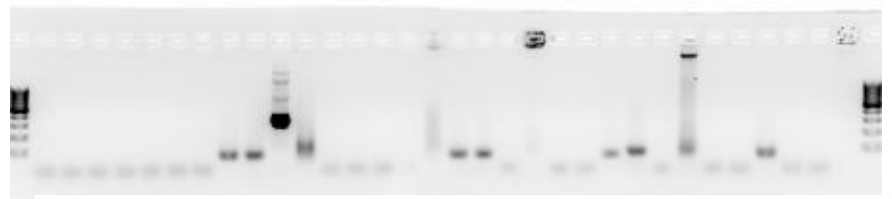
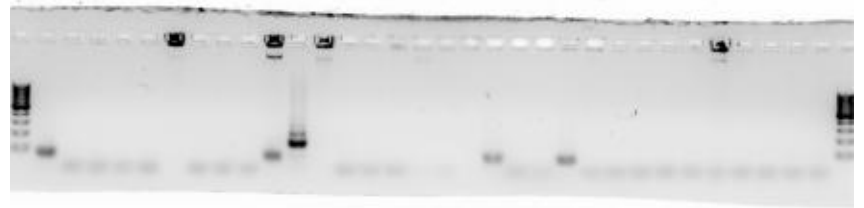
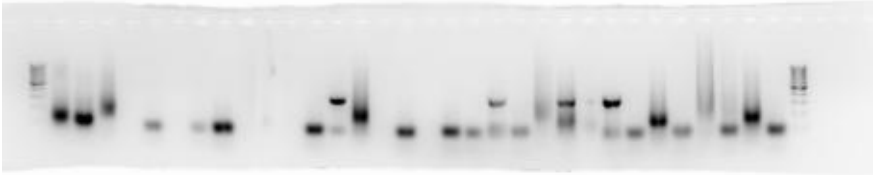
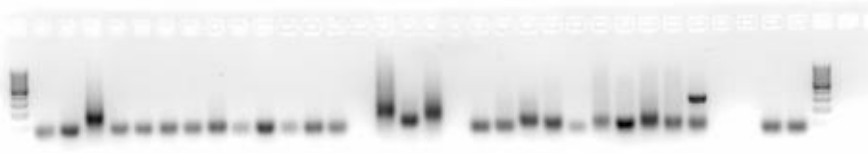
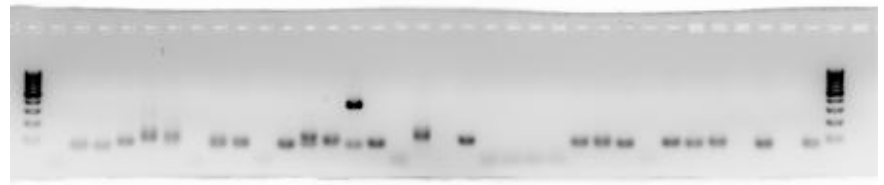
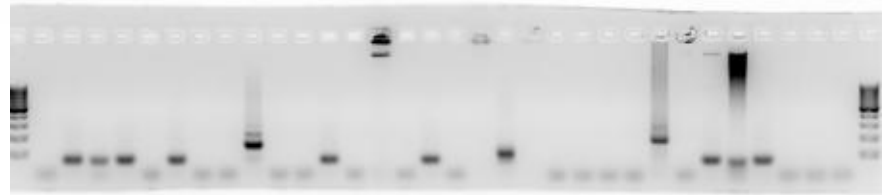
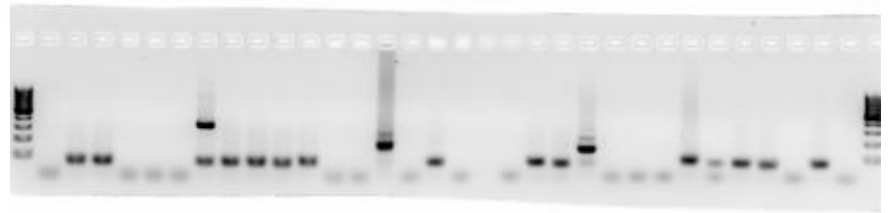
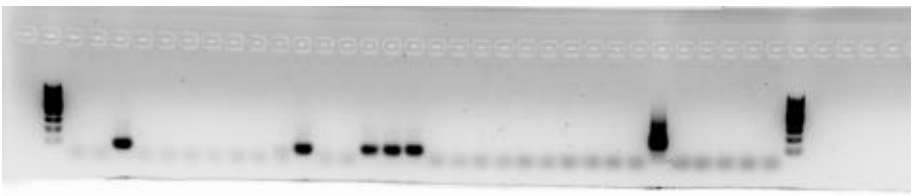

Low ΔΨm Aged Oocytes

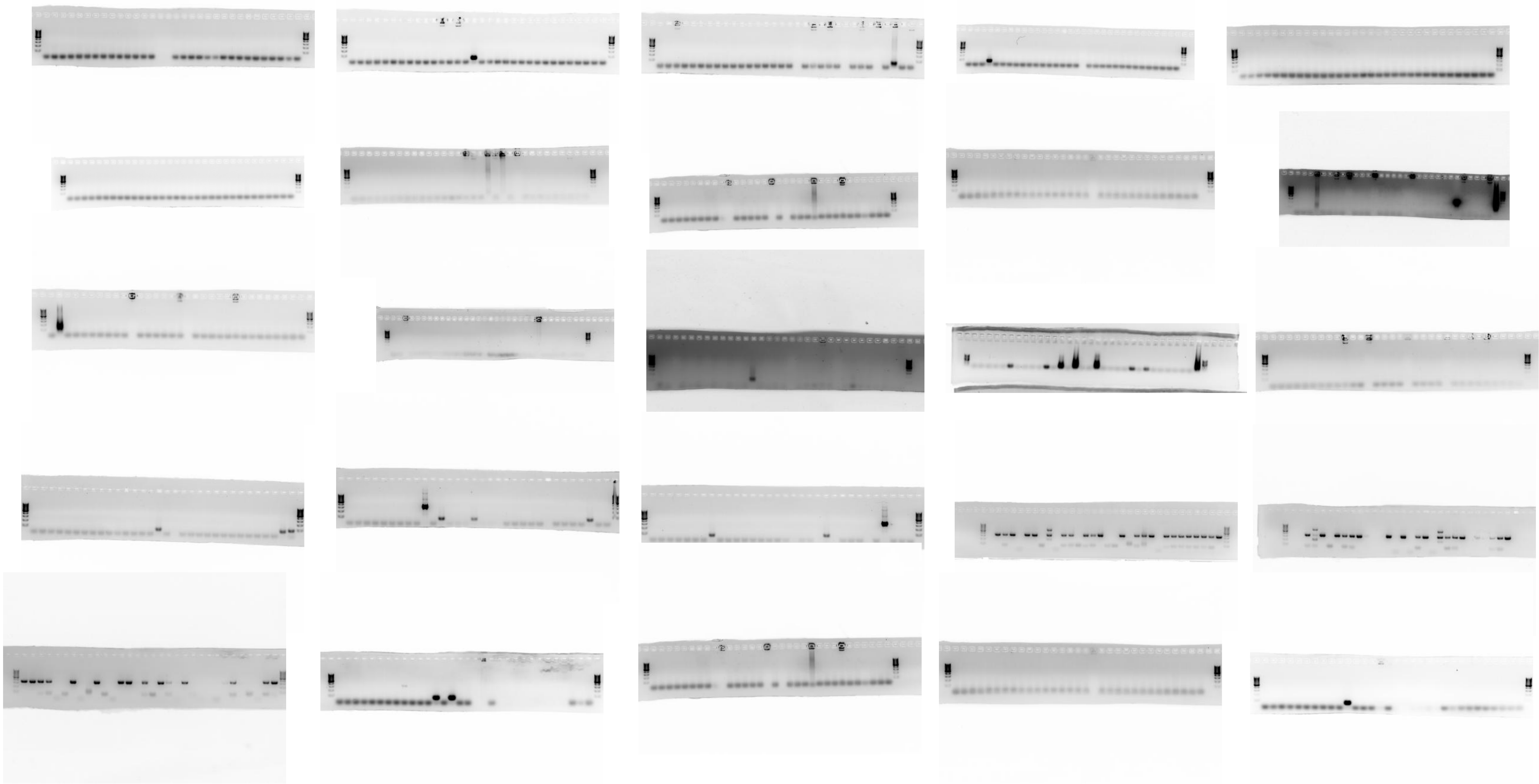

Low ΔΨm Aged Oocytes

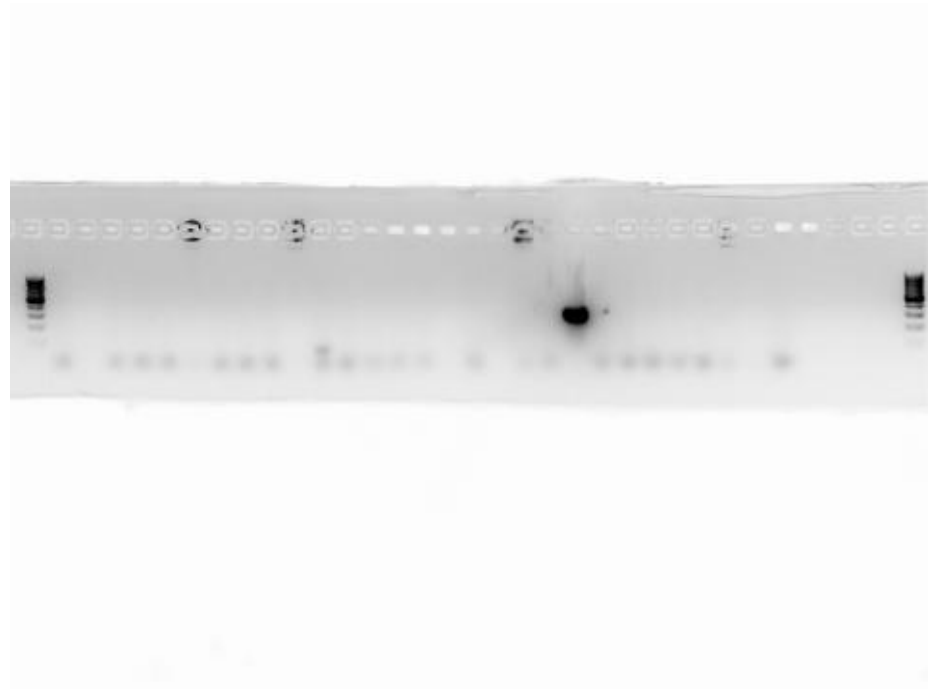
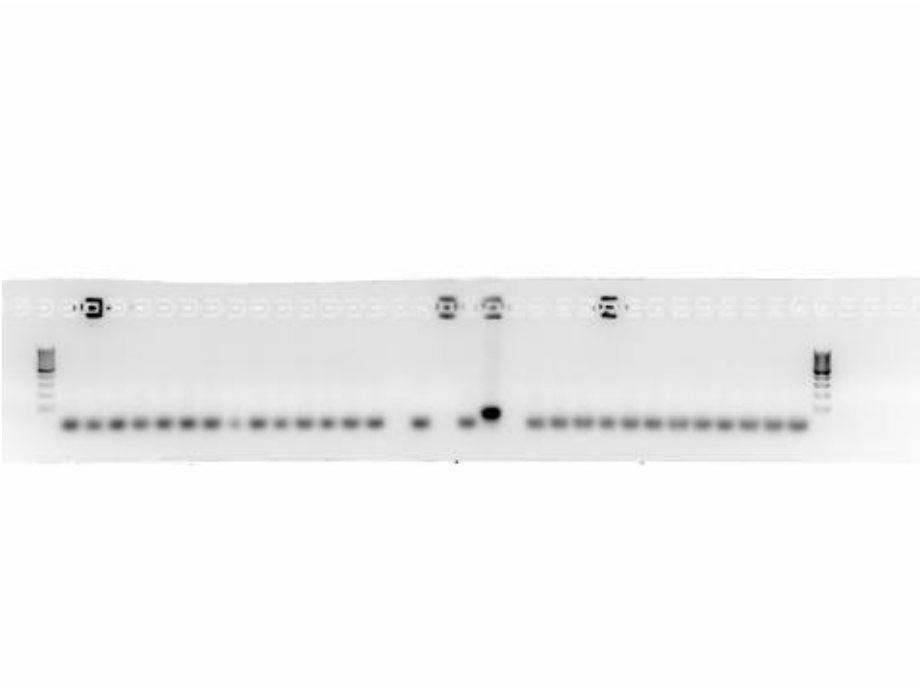
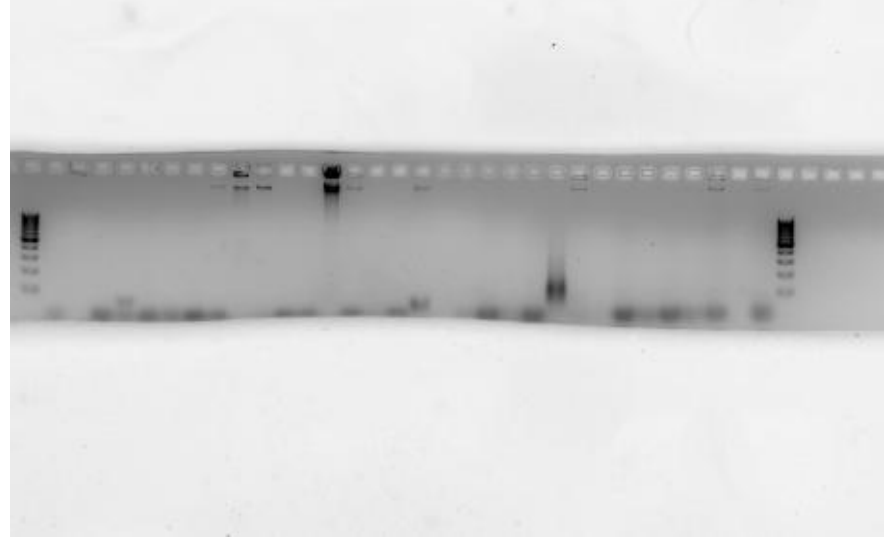
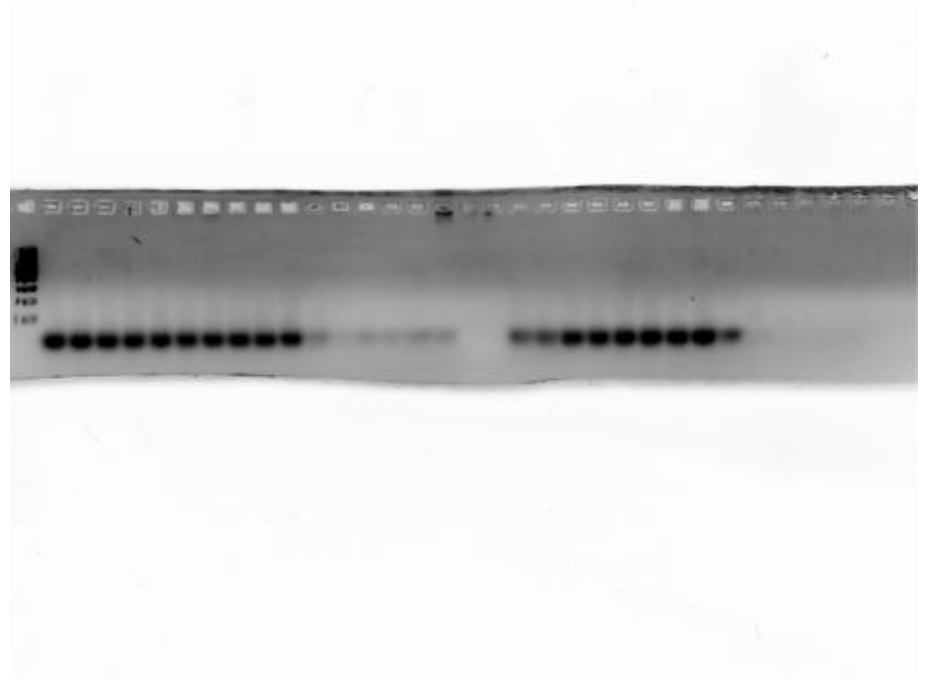
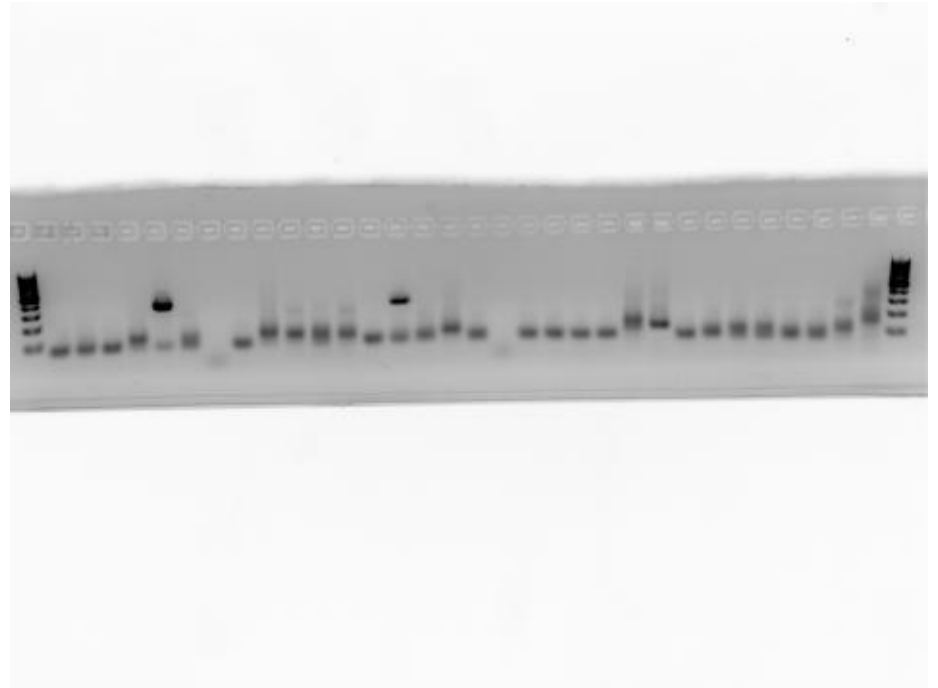

High ΔΨm Aged Oocytes

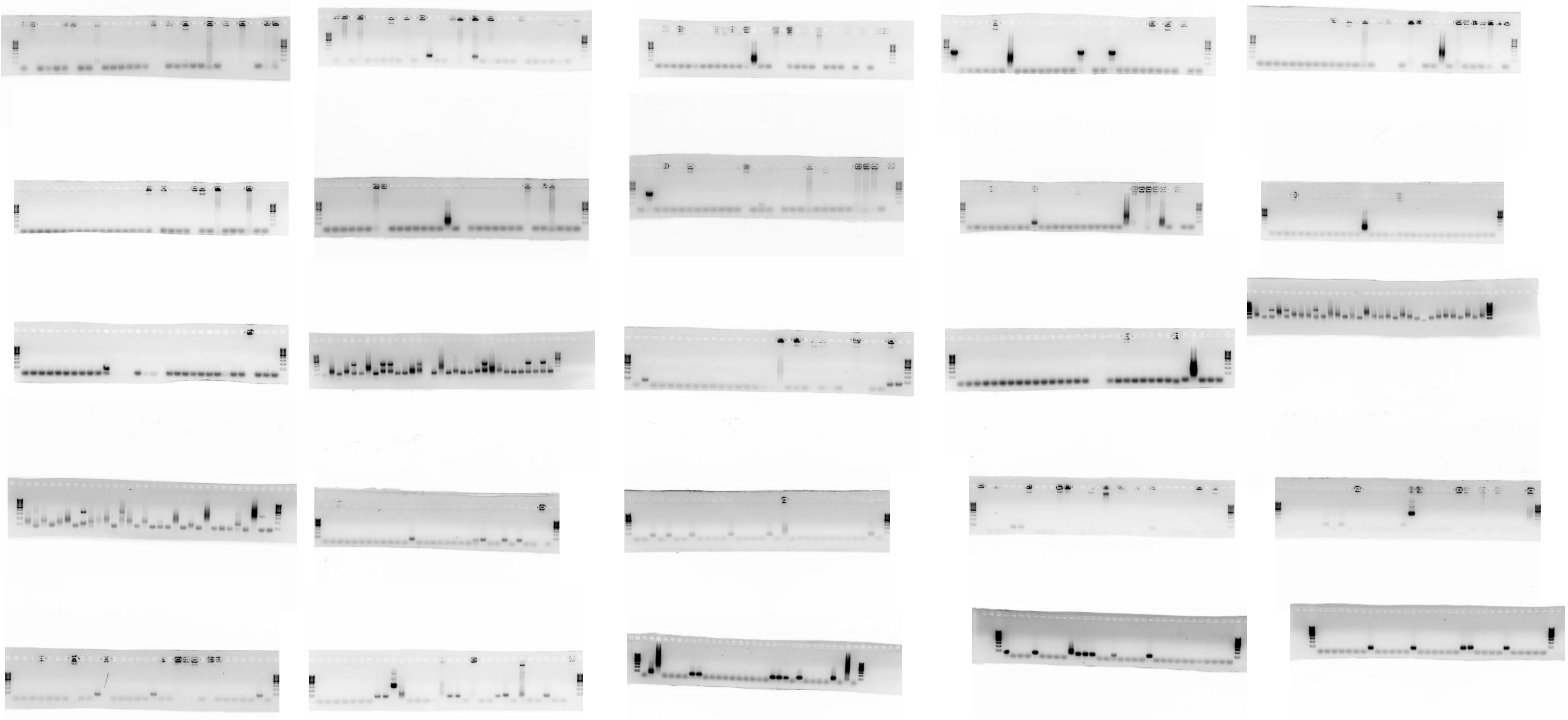

High ΔΨm Aged Oocytes

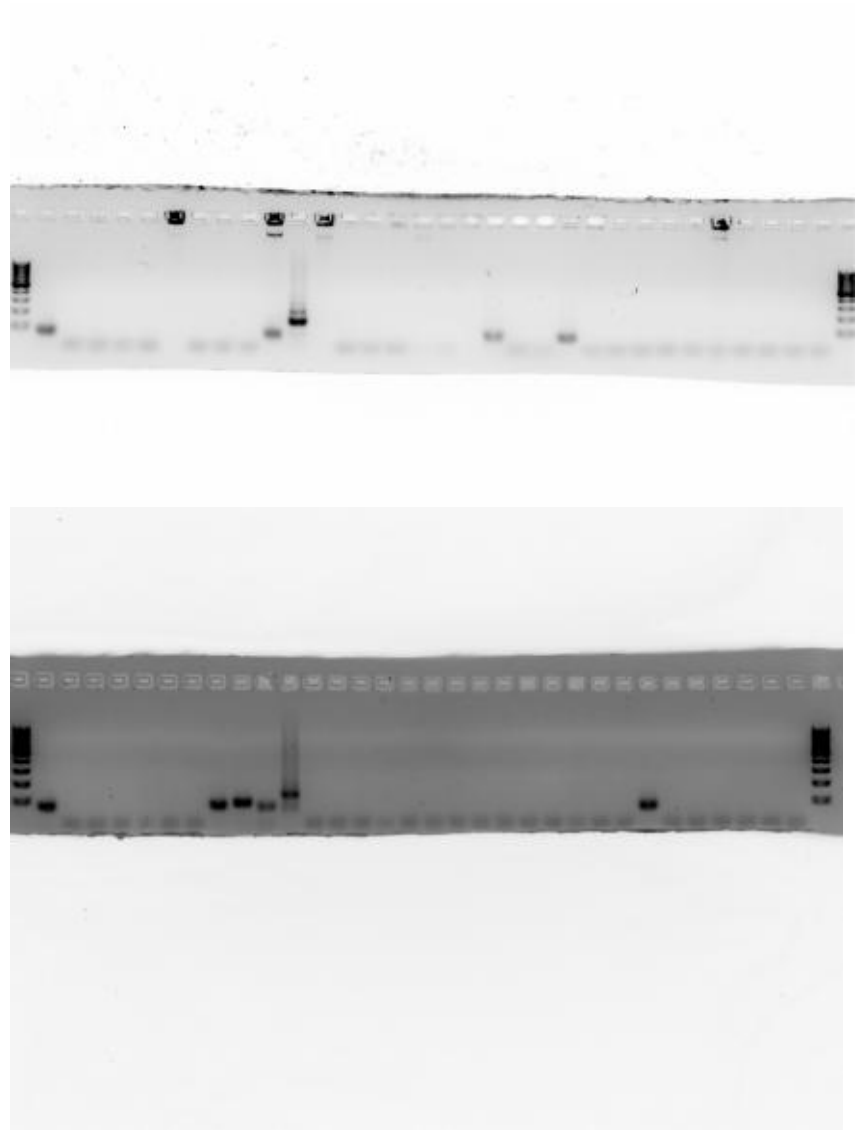
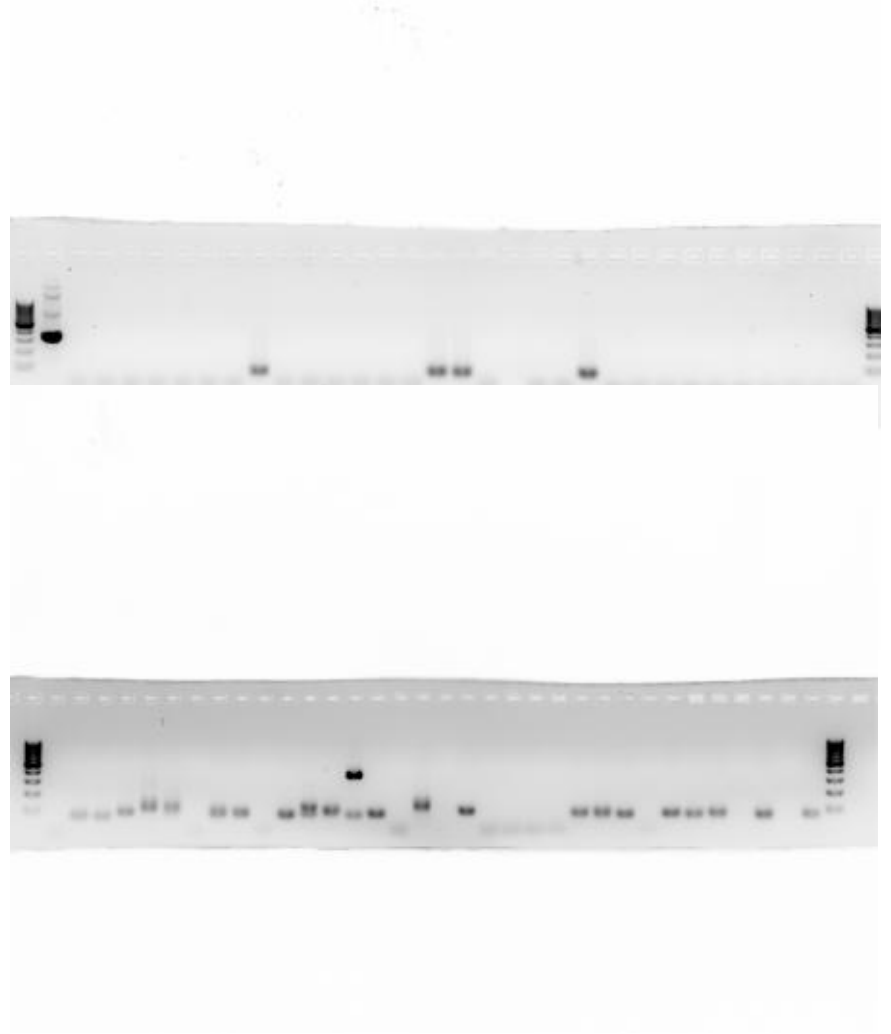
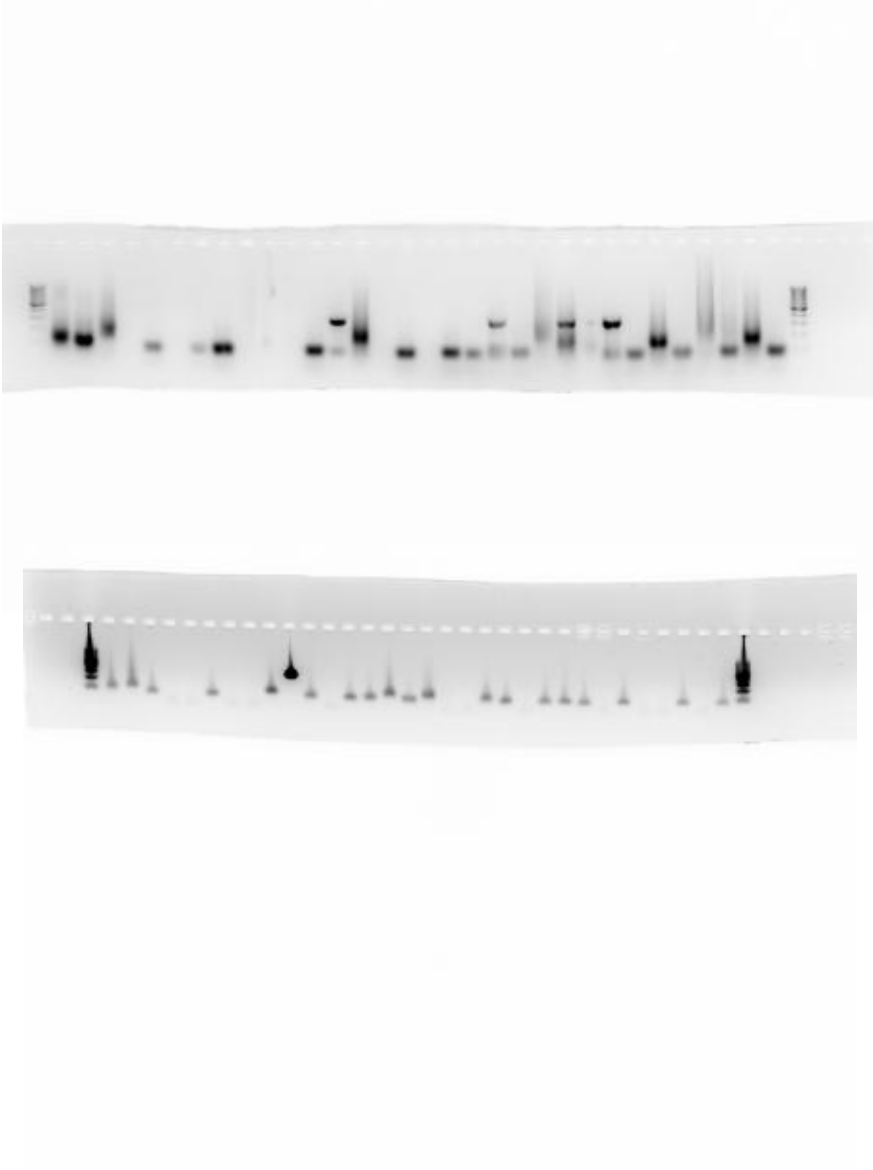
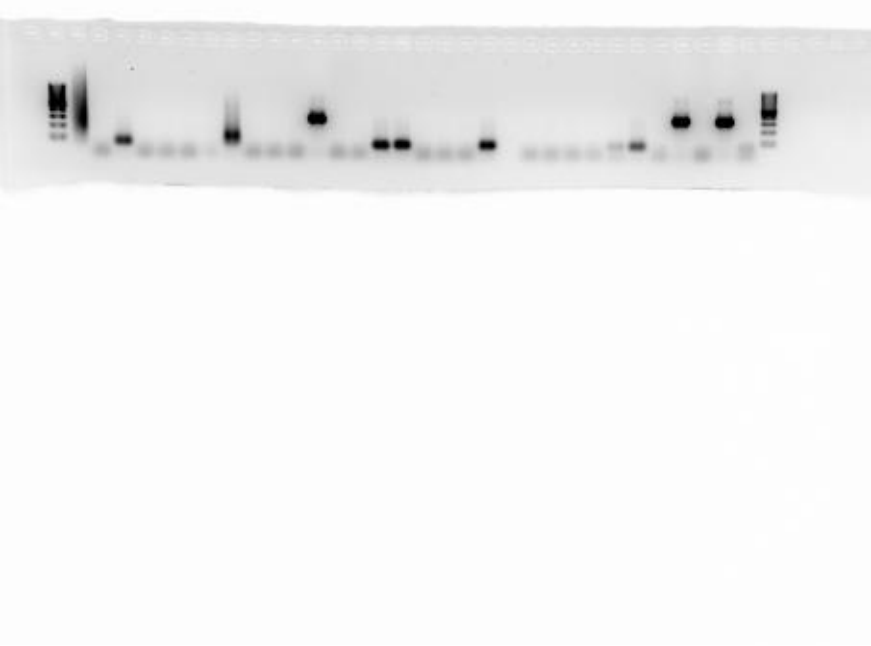
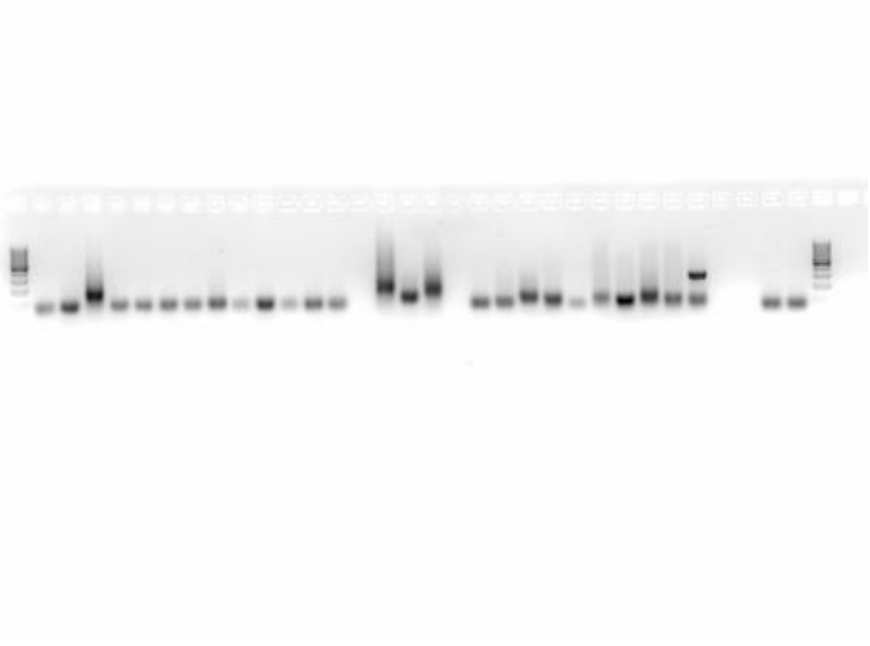

Supplement: Supplementary file 2 [file 13048_2026_2223_MOESM2_ESM.pdf]
