# Supplementary material for: Mitochondrial subpopulations in oocytes and cumulus cells exhibit distinct age-associated changes and selective plasticity in response to NMN supplementation
Source: J Ovarian Res. 2026 Aug 7;19:332. doi: 10.1186/s13048-026-02223-6 (PMC13732867; doi:10.1186/s13048-026-02223-6)

NMN Oocytes

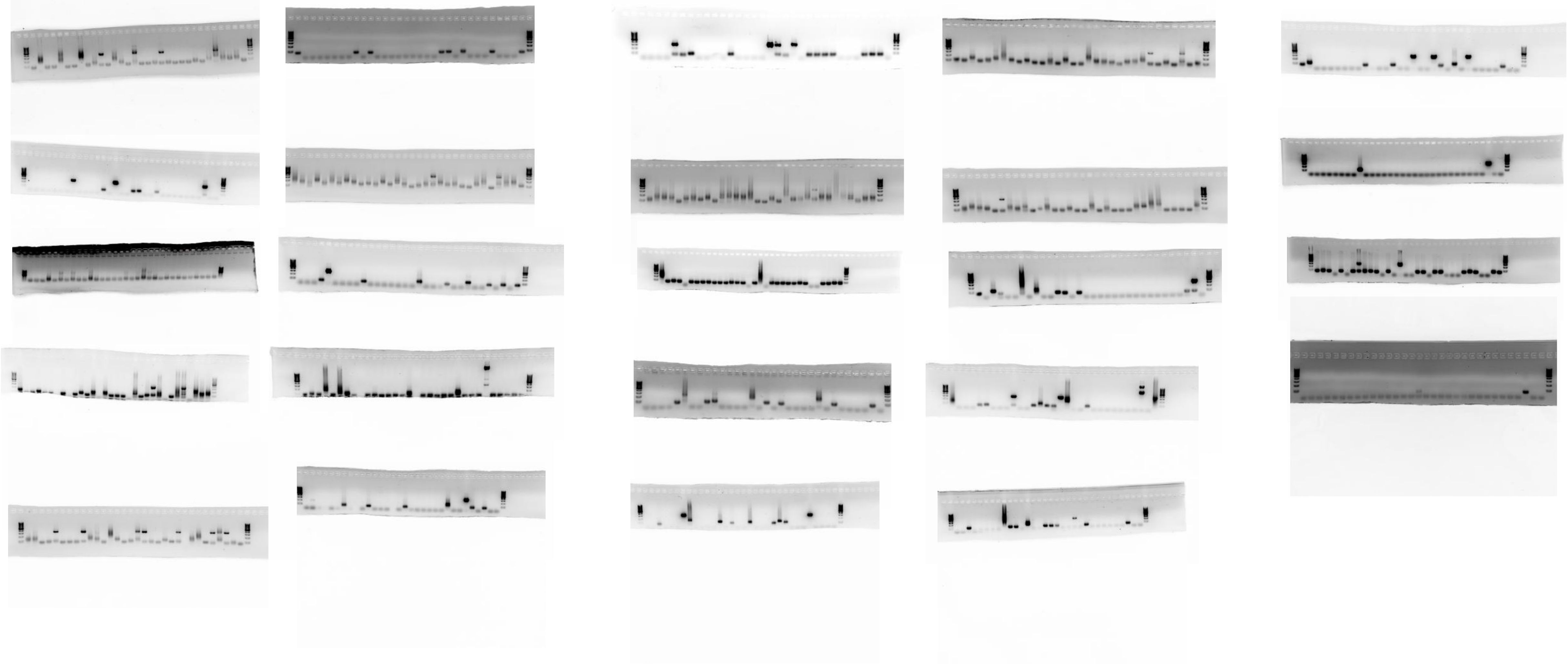

NMN Oocytes

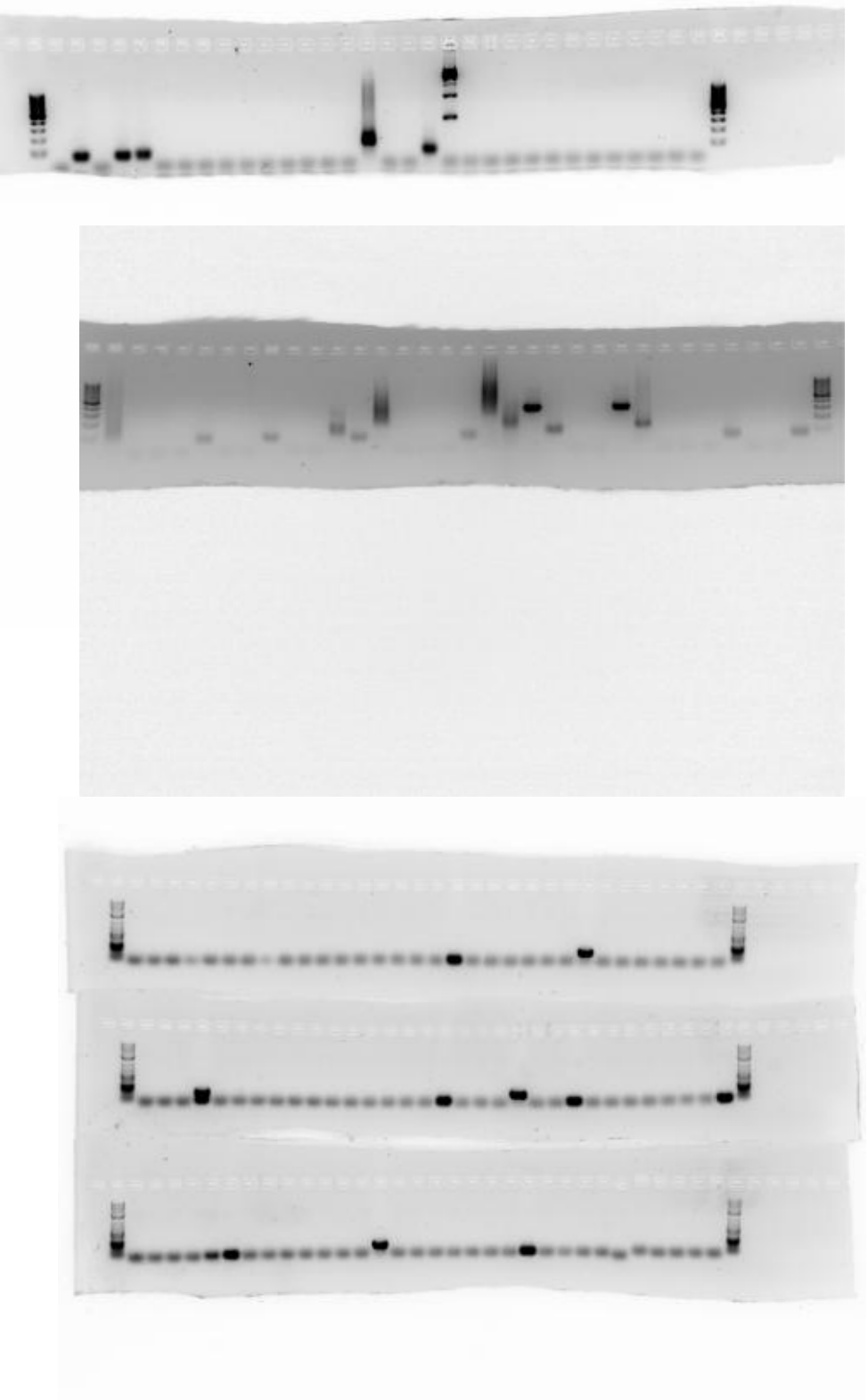

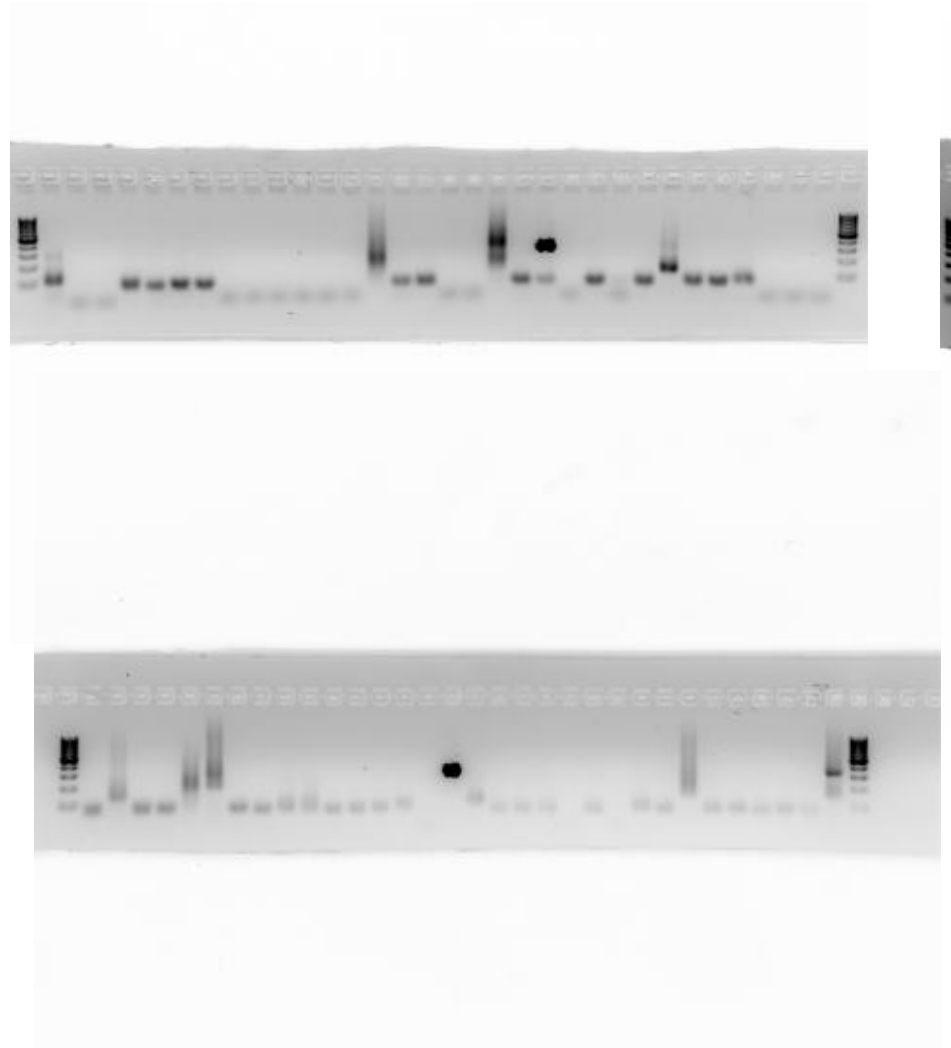

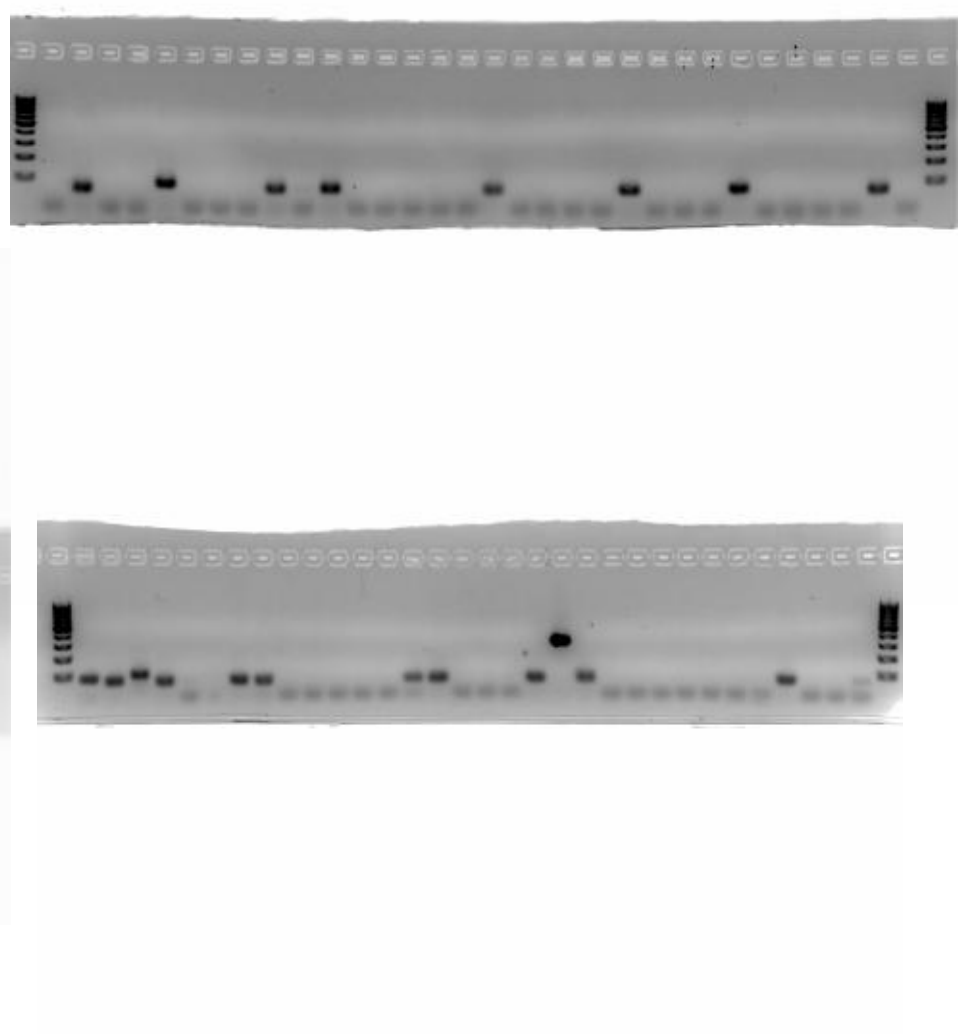

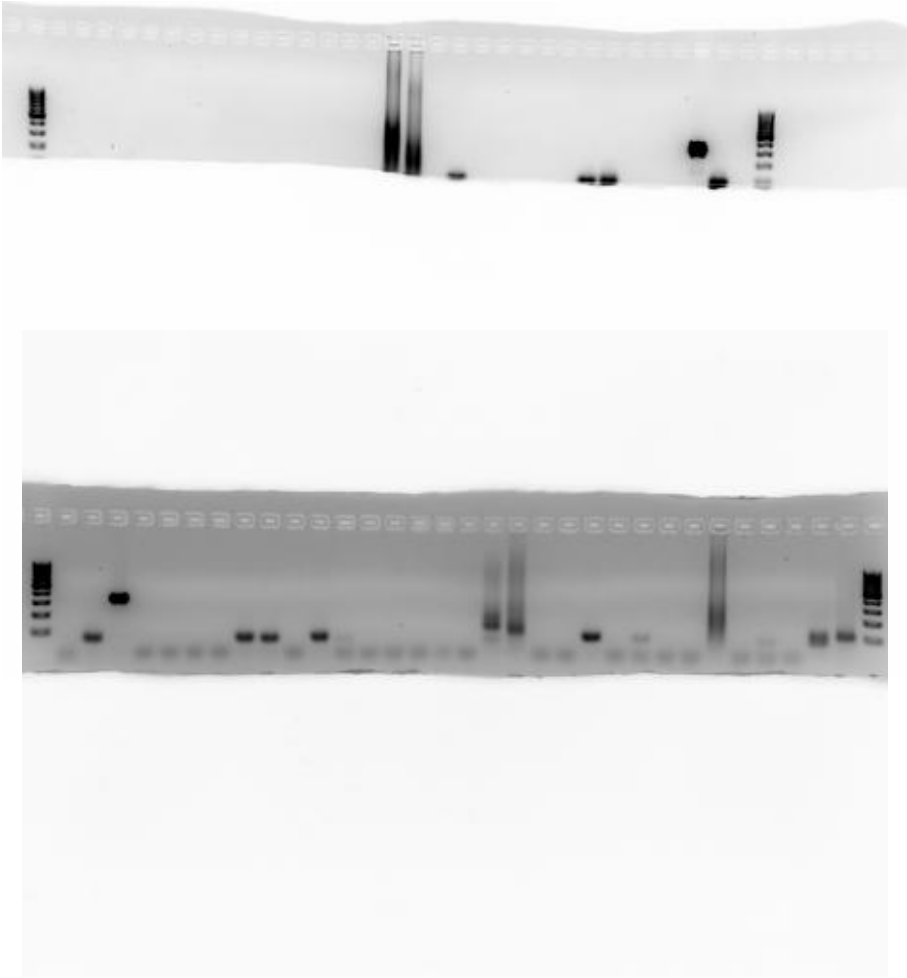

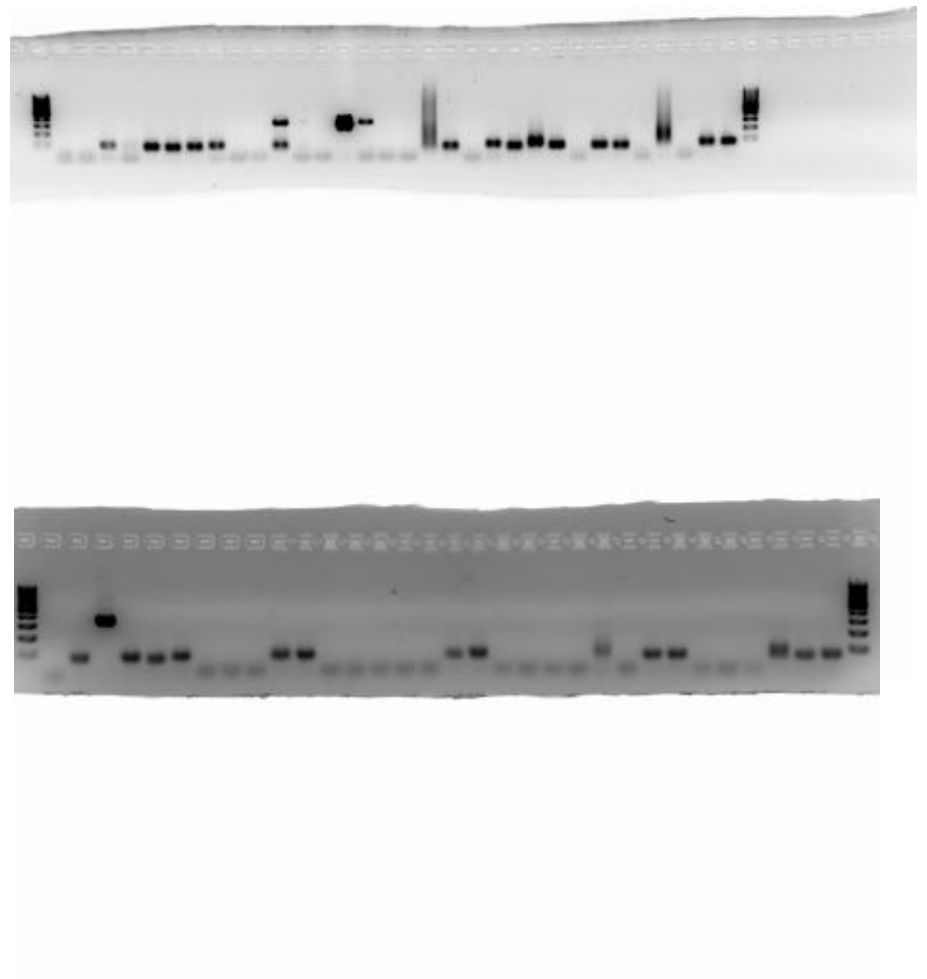

Small NMN Oocytes

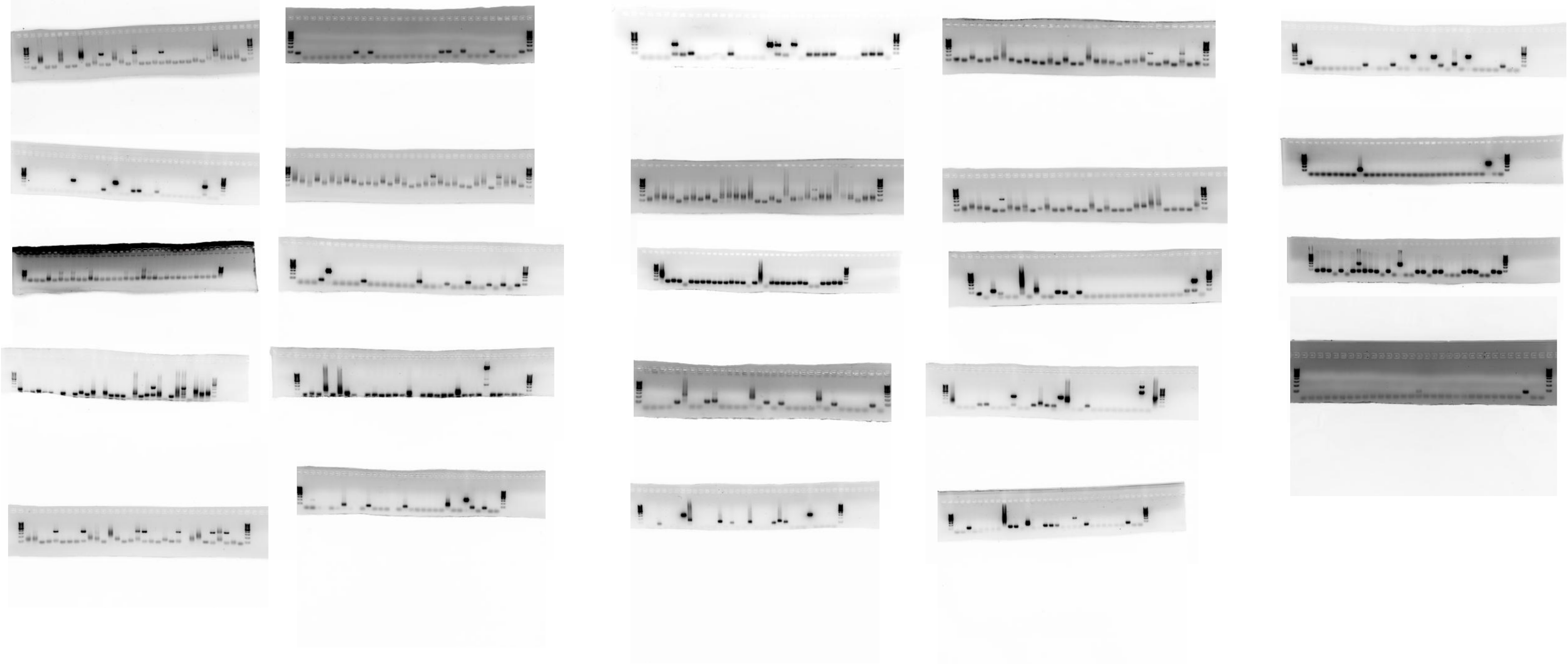

Large NMN Oocytes

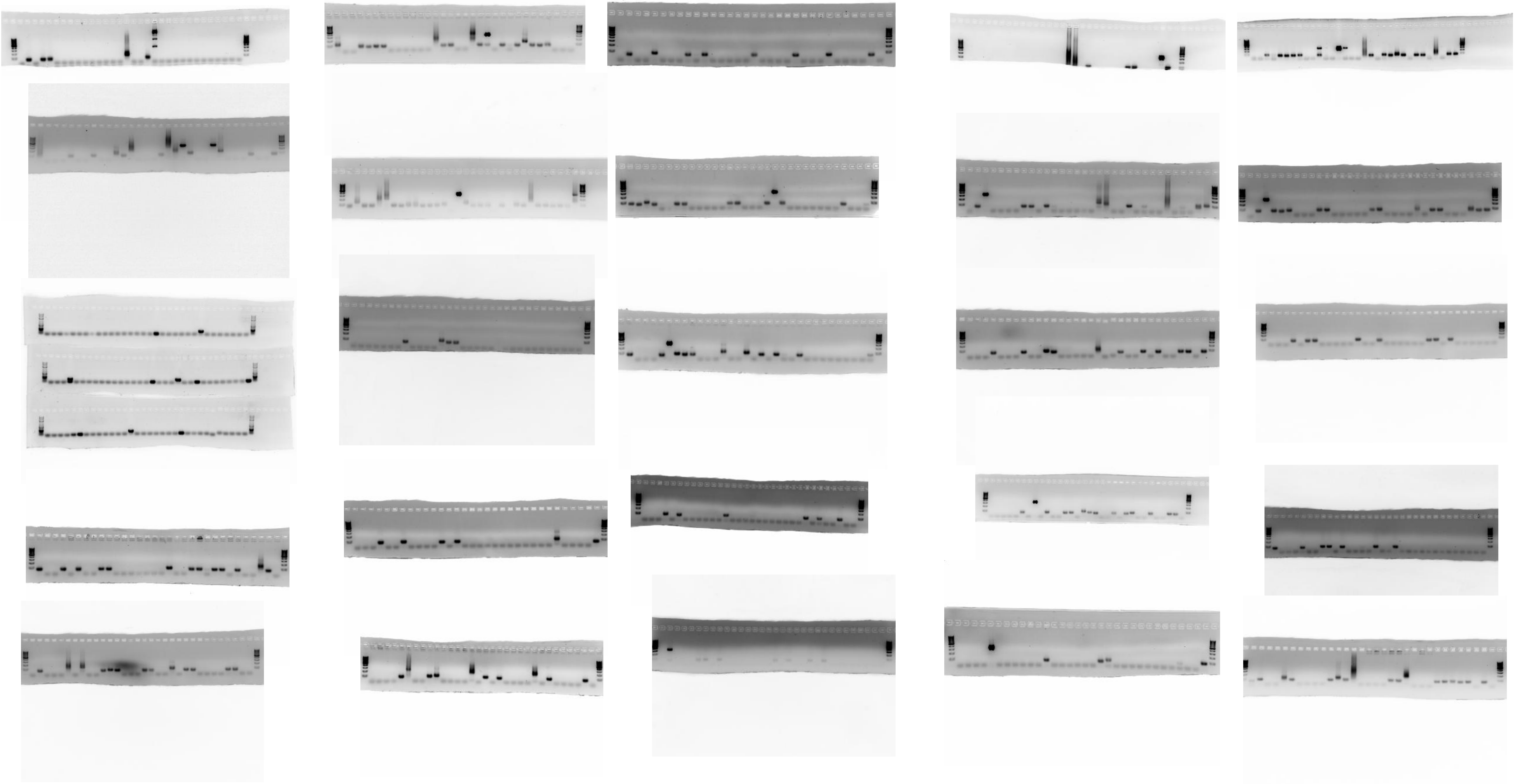

Low ΔΨm NMN Oocytes

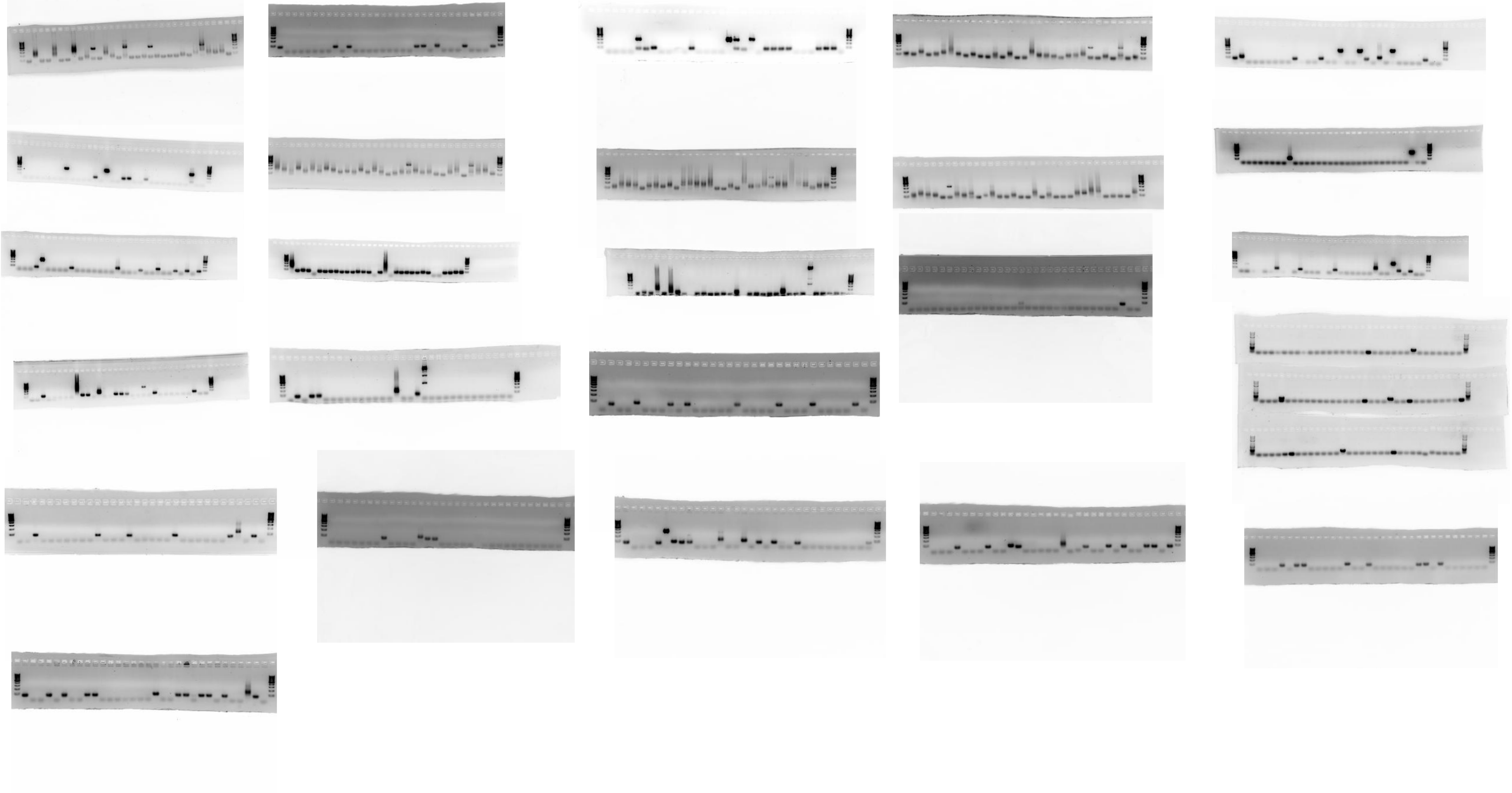

High ΔΨm NMN Oocytes

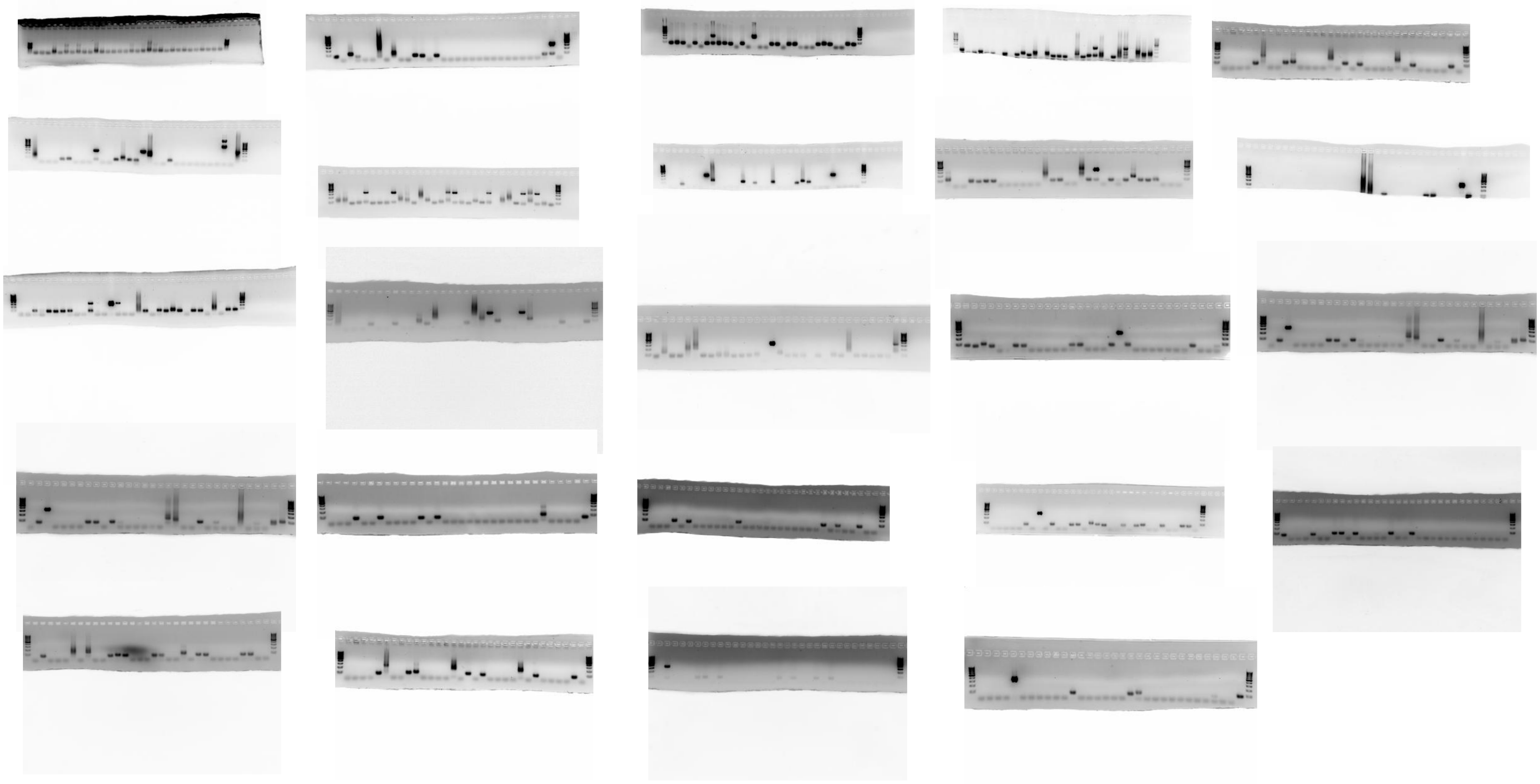

Supplement: Supplementary file 4 [file 13048_2026_2223_MOESM4_ESM.pdf]
