# Supplementary material for: Mitochondrial subpopulations in oocytes and cumulus cells exhibit distinct age-associated changes and selective plasticity in response to NMN supplementation
Source: J Ovarian Res. 2026 Aug 7;19:332. doi: 10.1186/s13048-026-02223-6 (PMC13732867; doi:10.1186/s13048-026-02223-6)

Young Cumulus Cells

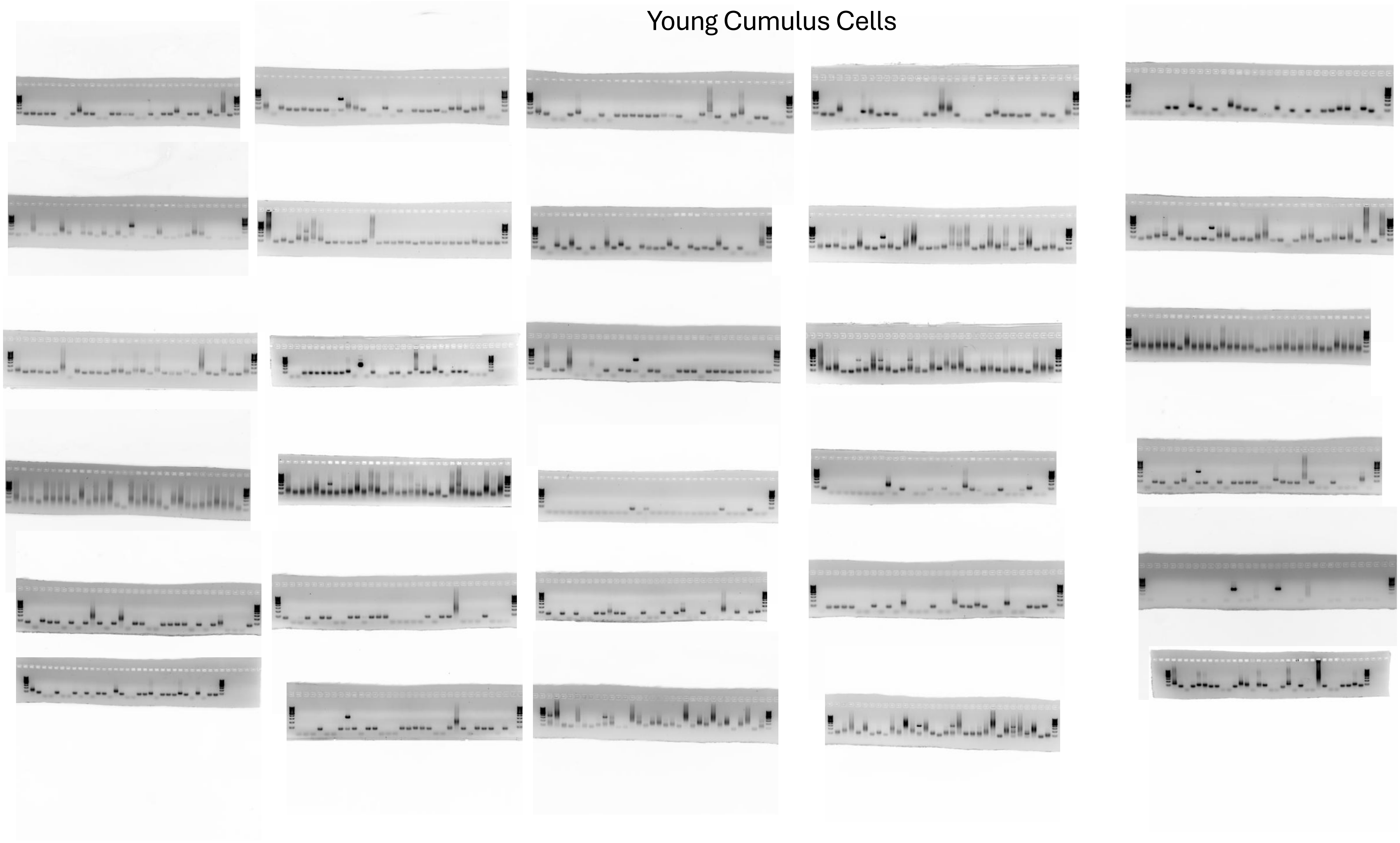

Young Cumulus Cells

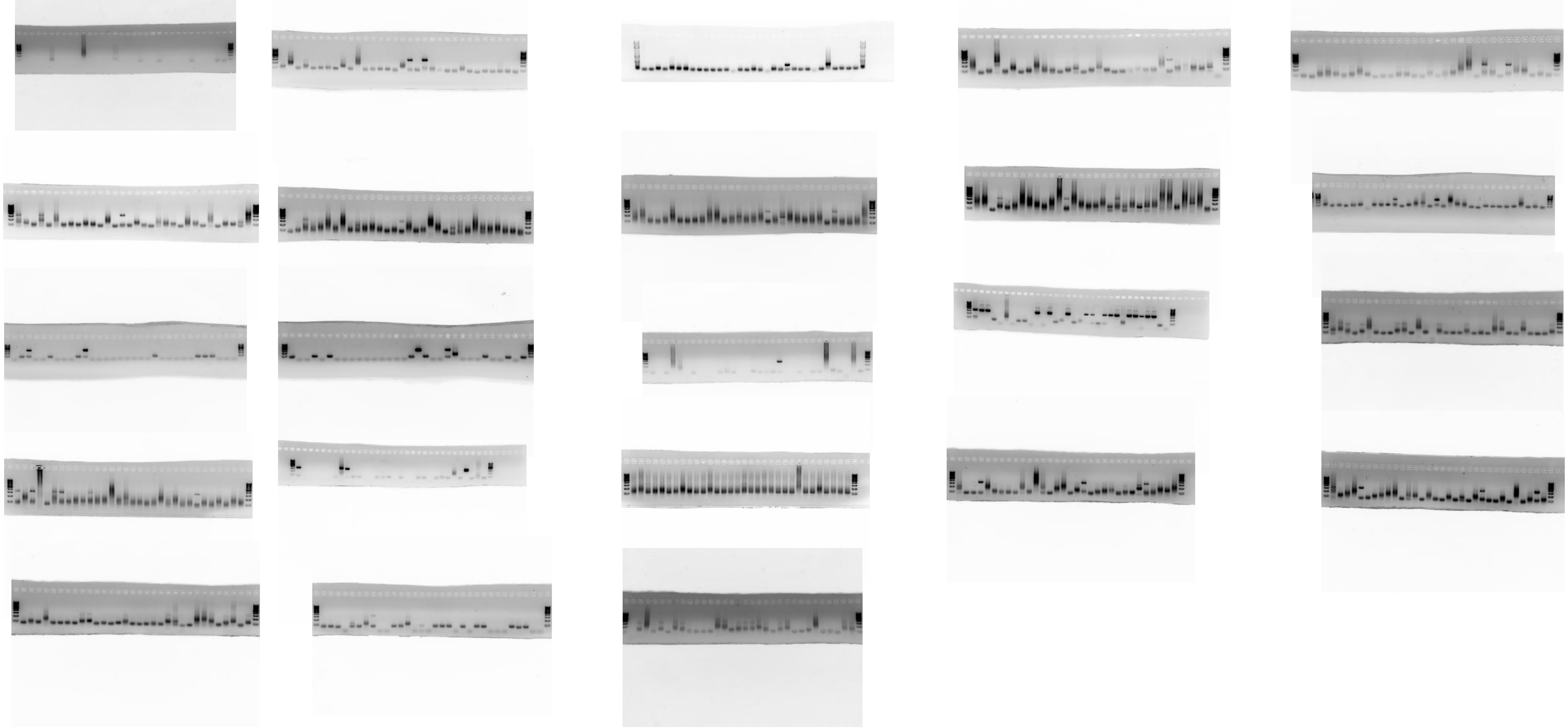

Small Young Cumulus Cells

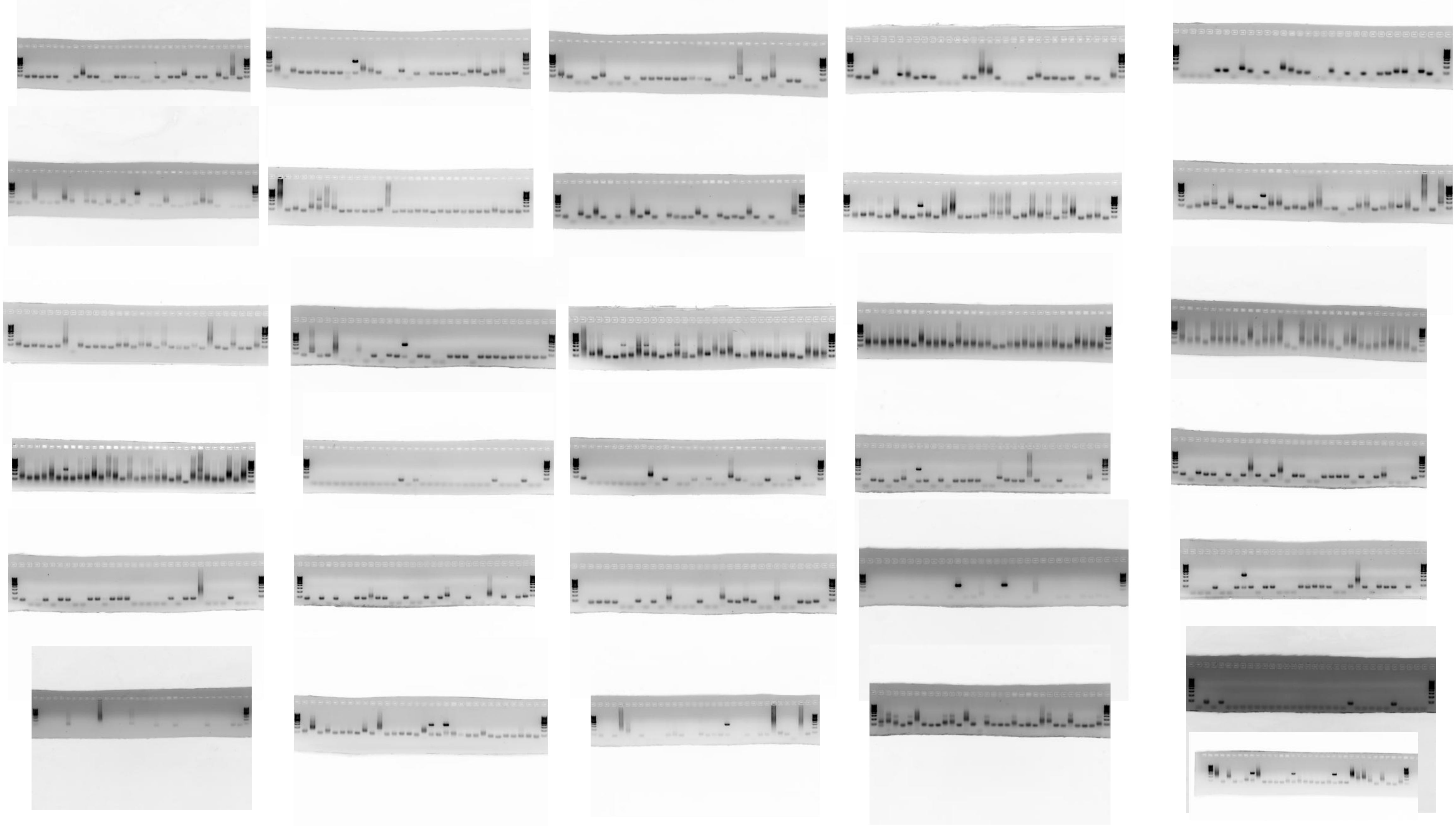

Large Young Cumulus Cells

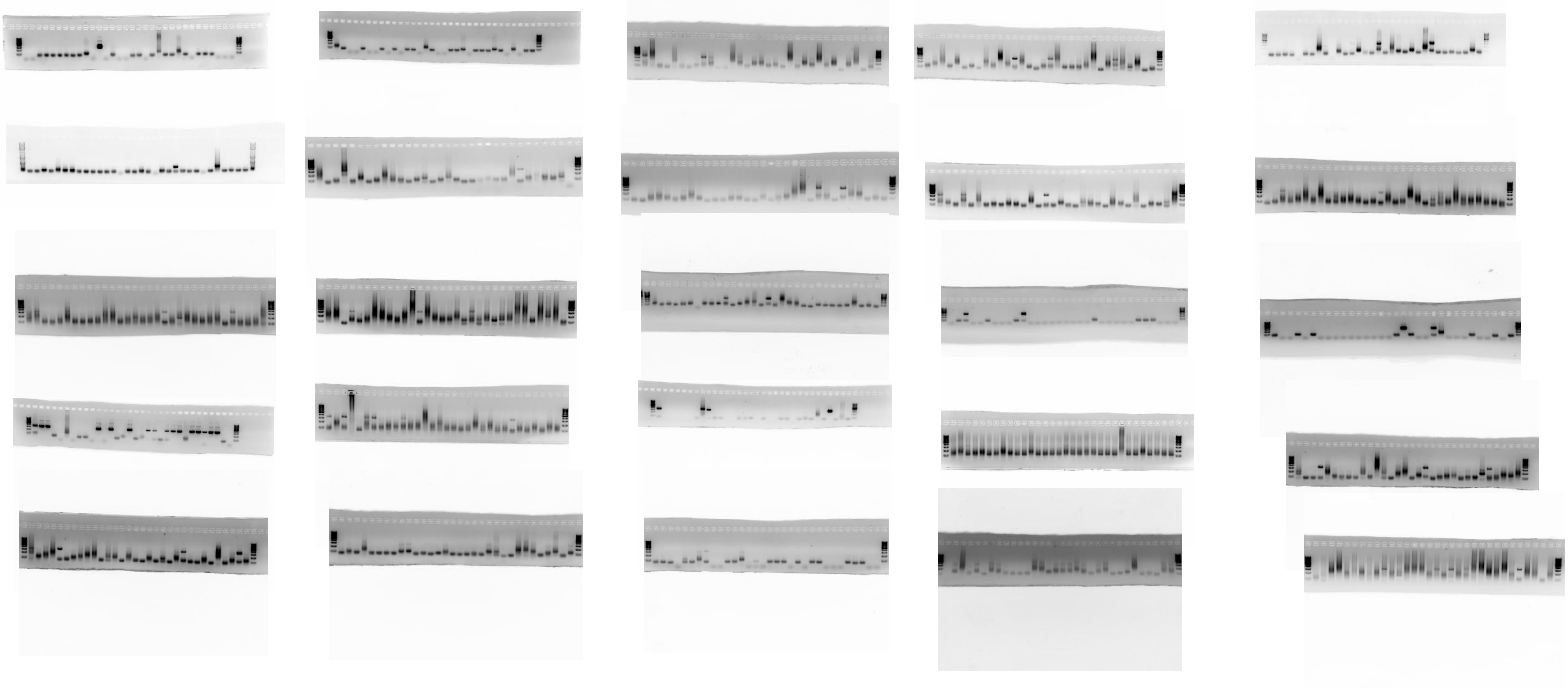

Large Young Cumulus Cells

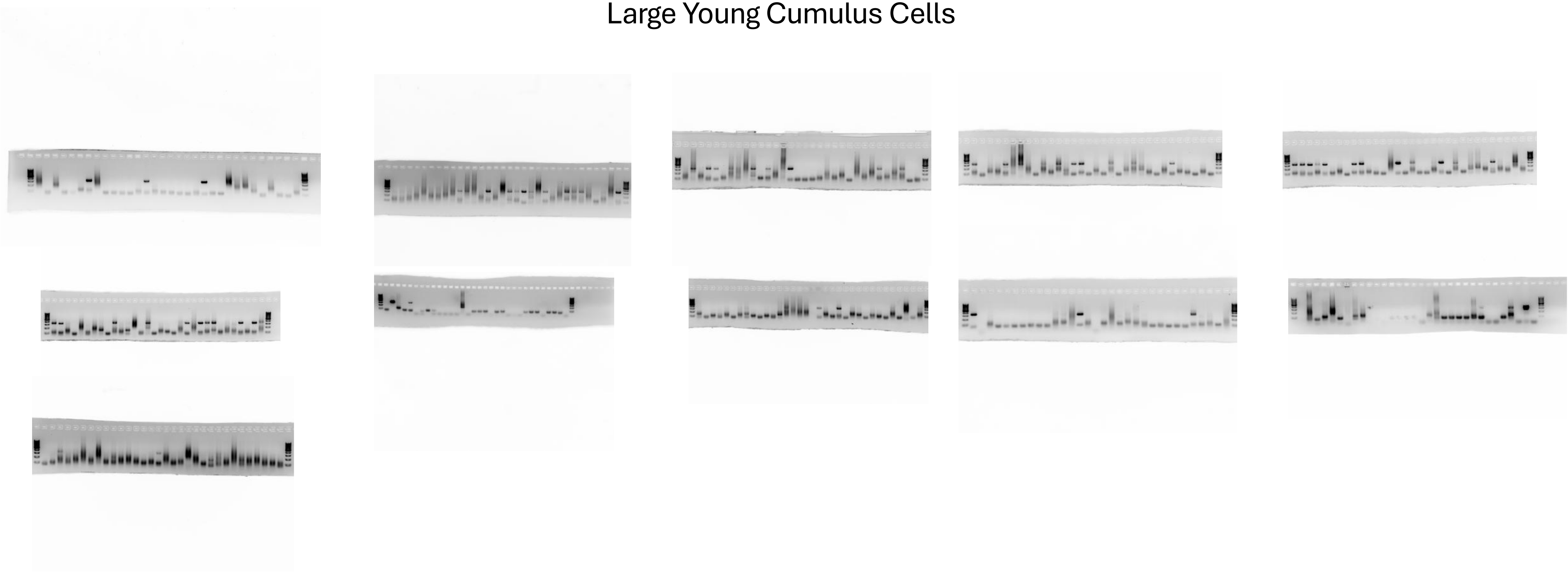

Low ΔΨm Young Cumulus Cells

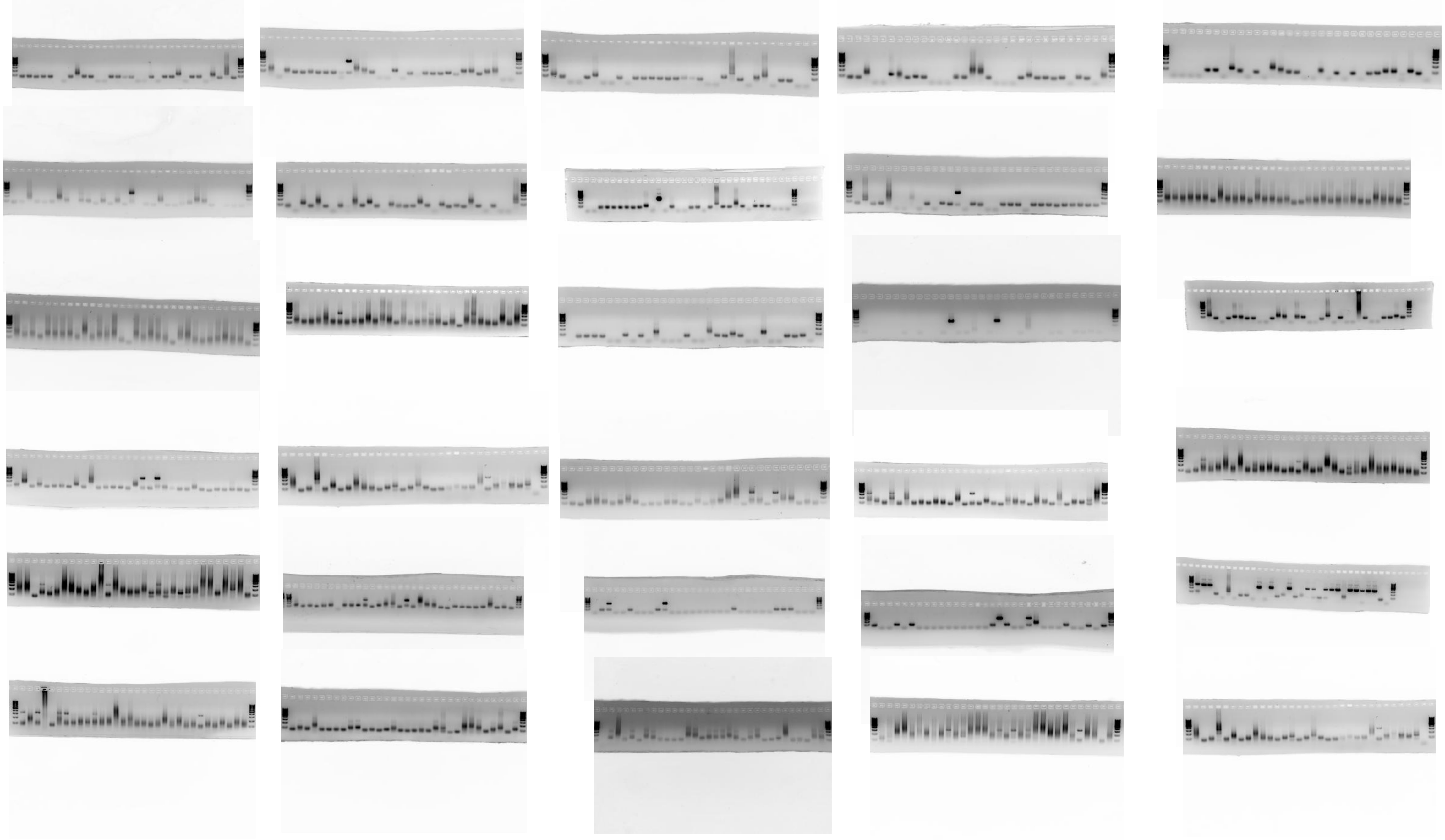

Low ΔΨm Young Cumulus Cells

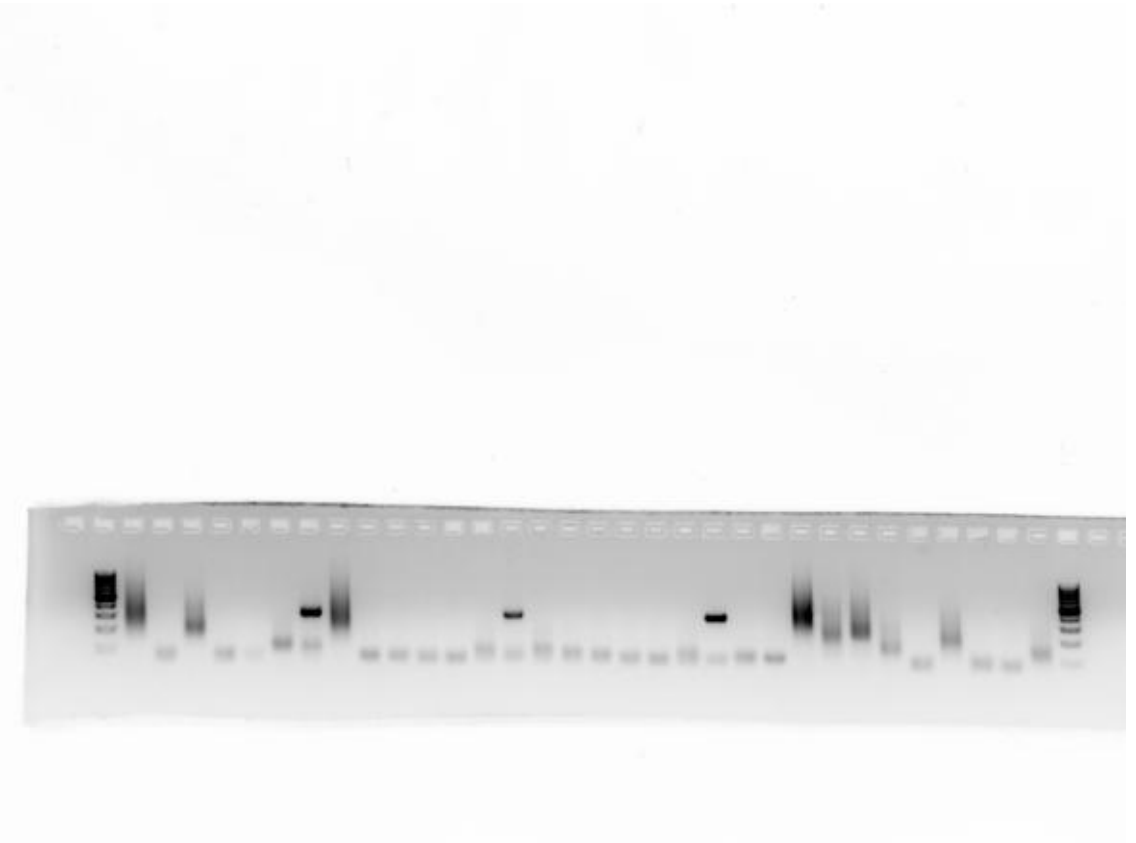

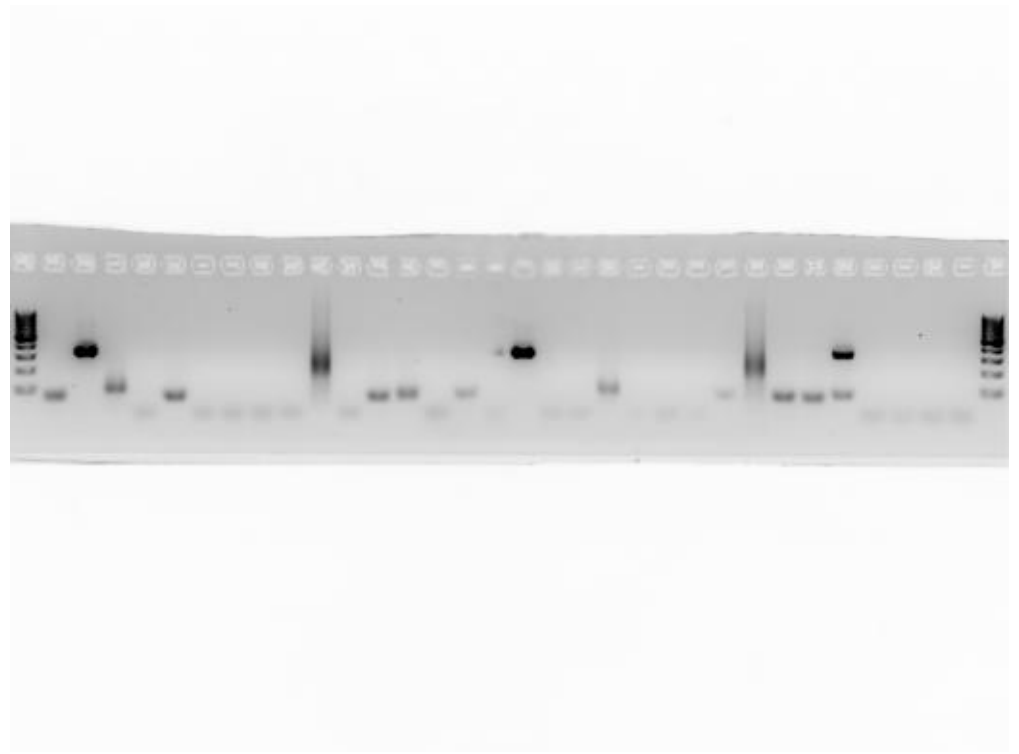

High ΔΨm Young Cumulus Cells

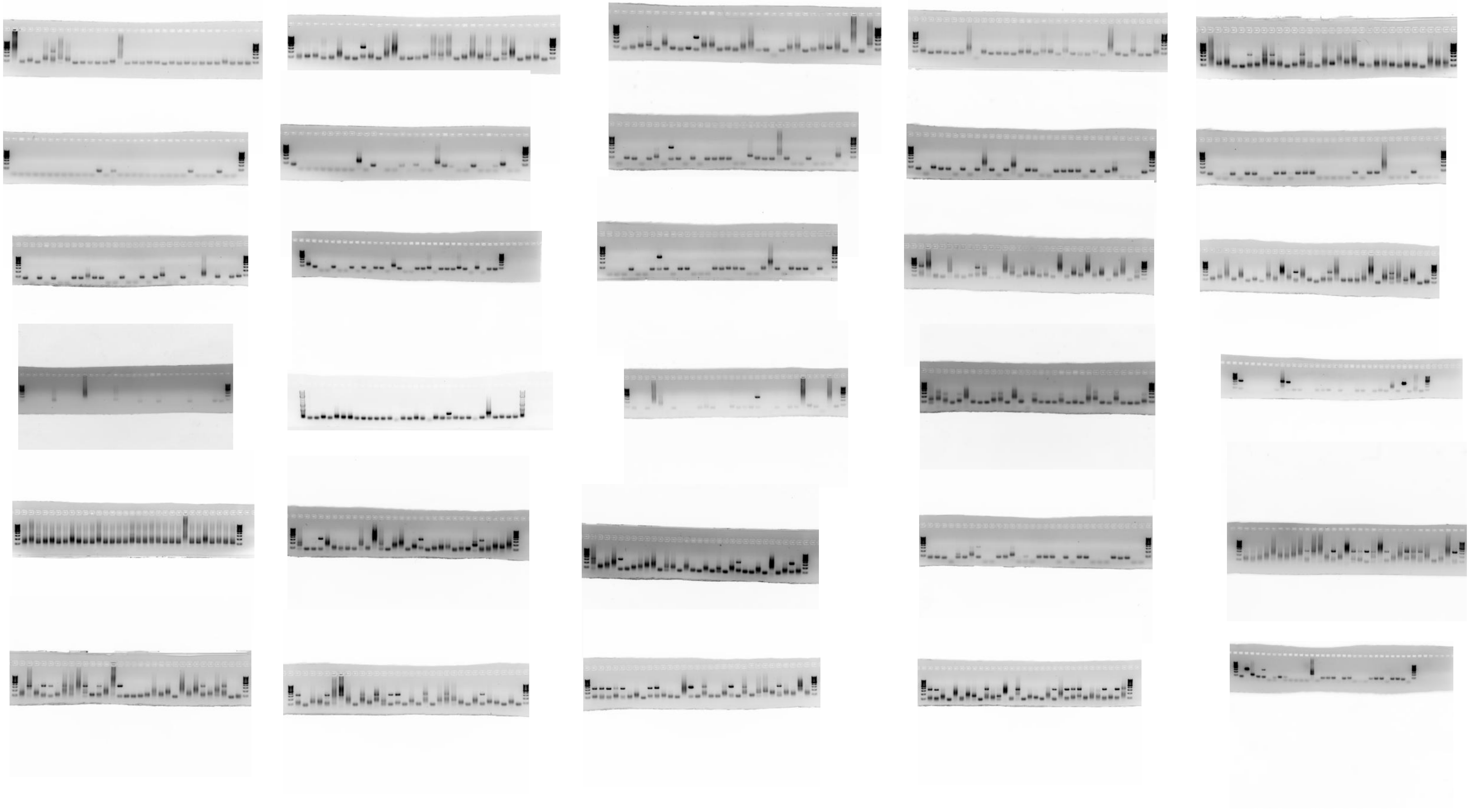

High ΔΨm Young Cumulus Cells

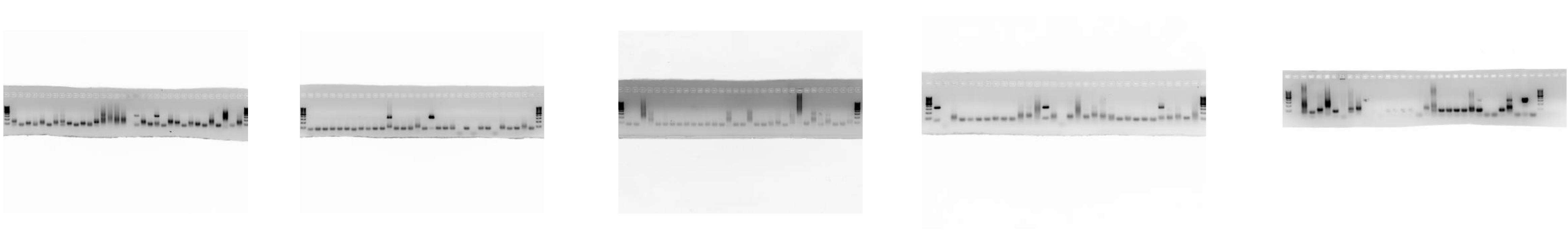

Supplement: Supplementary file 5 [file 13048_2026_2223_MOESM5_ESM.pdf]
