# Supplementary material for: Mitochondrial subpopulations in oocytes and cumulus cells exhibit distinct age-associated changes and selective plasticity in response to NMN supplementation
Source: J Ovarian Res. 2026 Aug 7;19:332. doi: 10.1186/s13048-026-02223-6 (PMC13732867; doi:10.1186/s13048-026-02223-6)

Young Oocytes

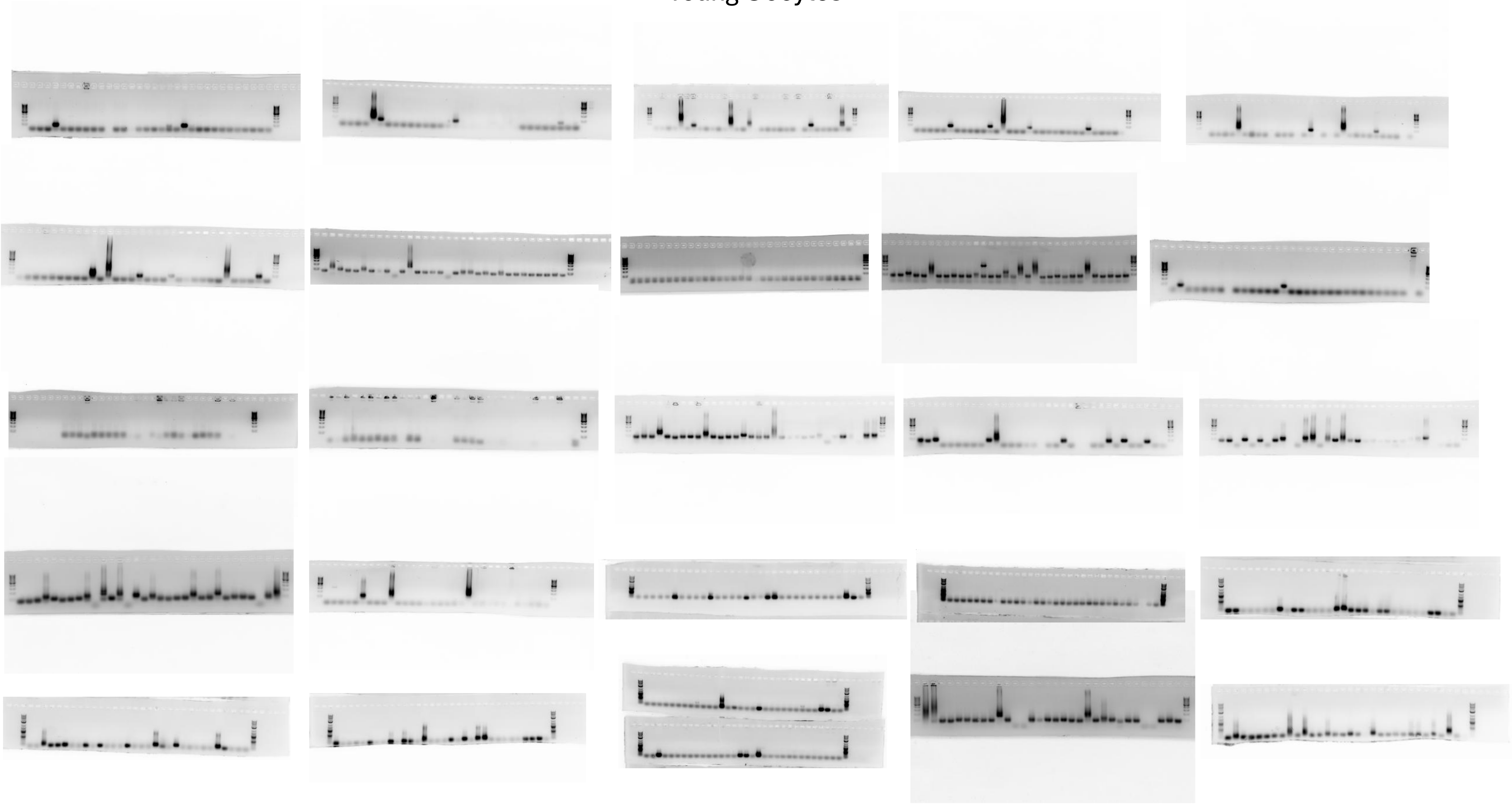

Young Oocytes

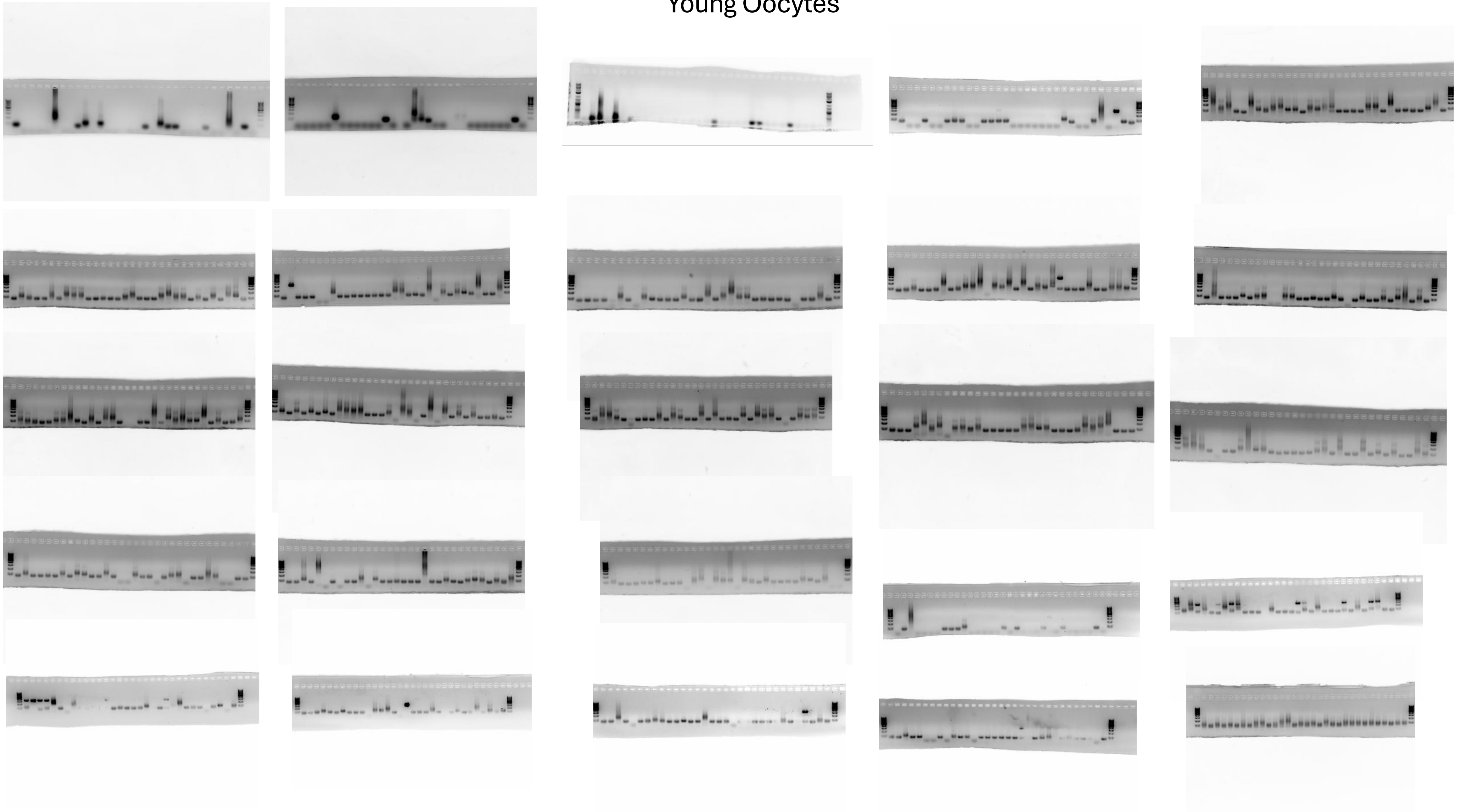

Young Oocytes

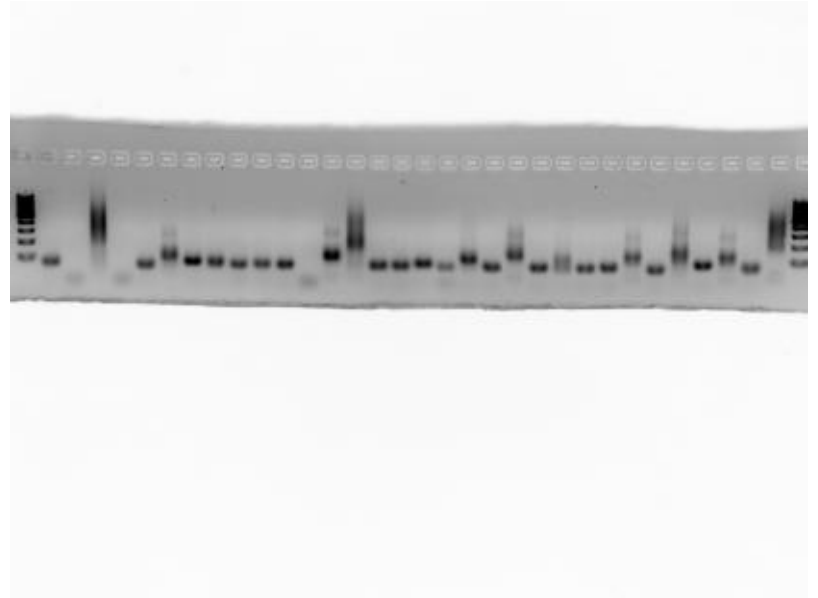

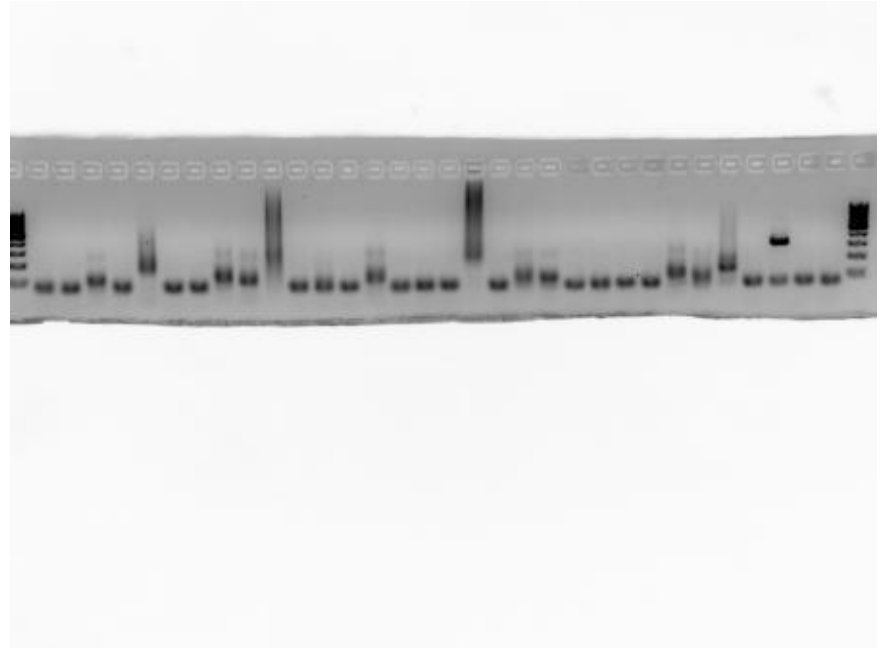

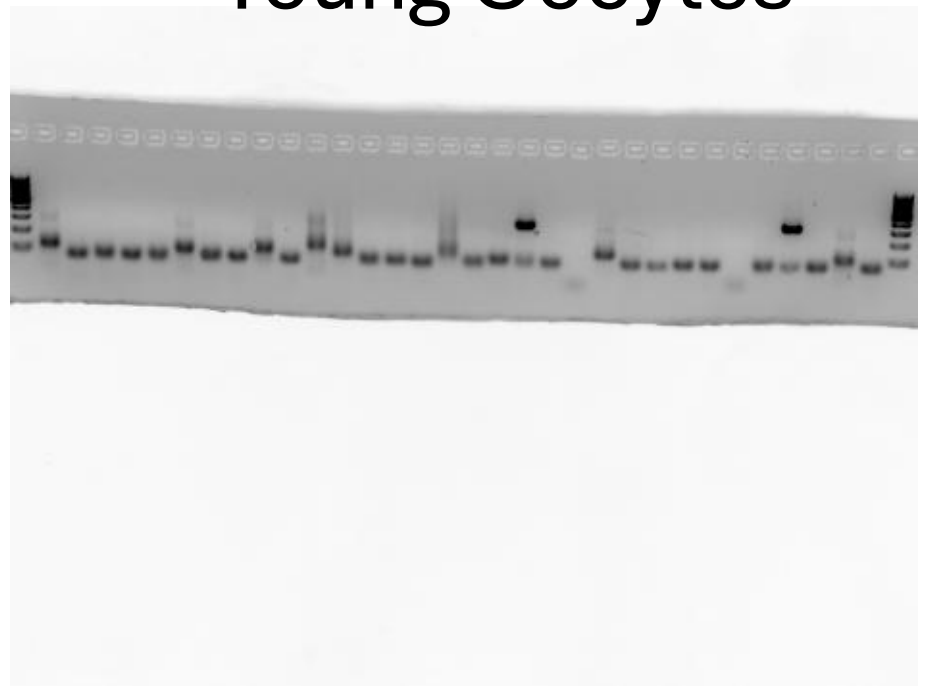

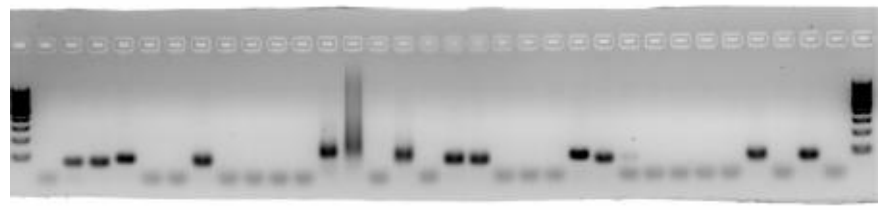

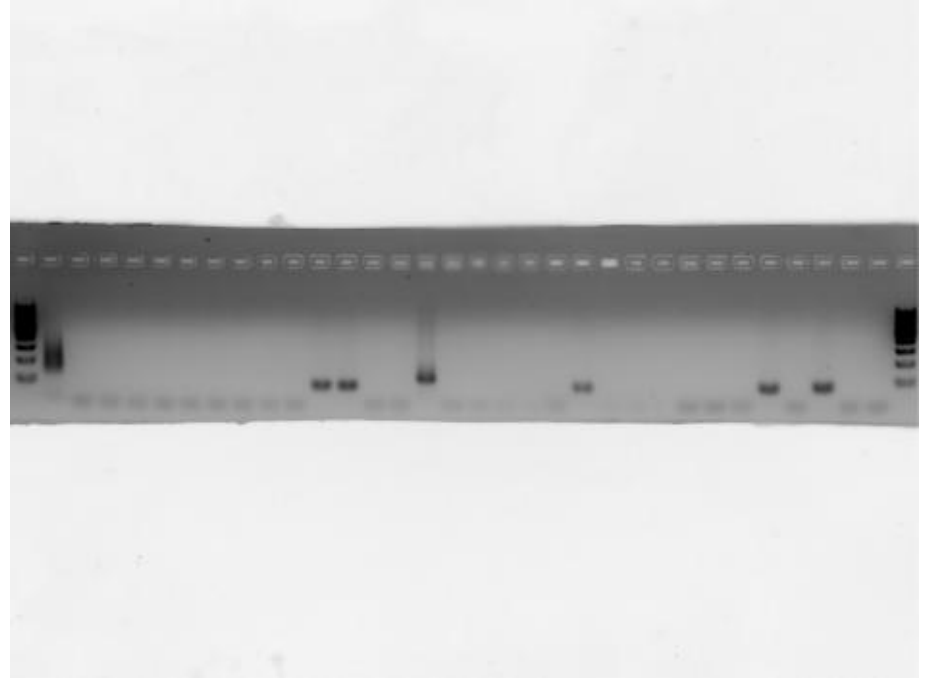

Small Young Oocytes

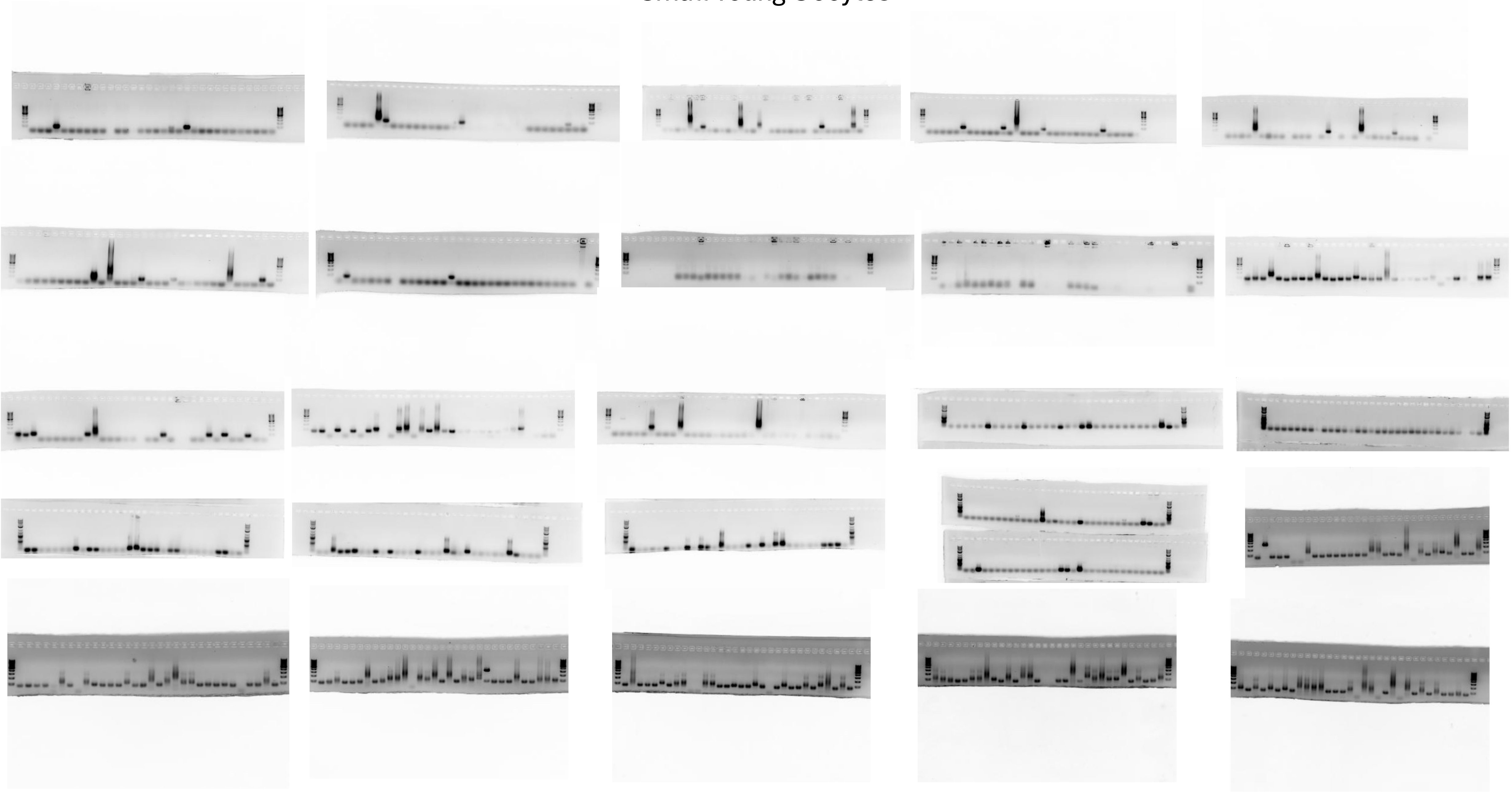

Small Young Oocytes

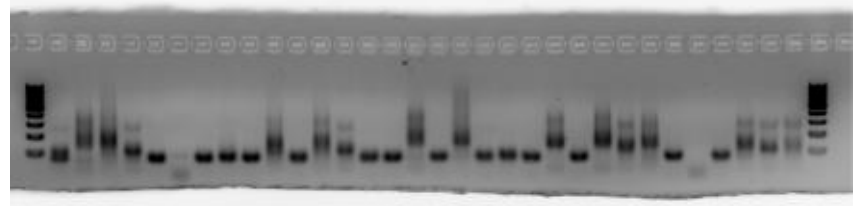
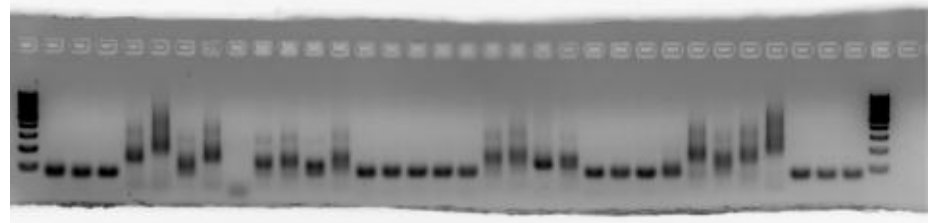
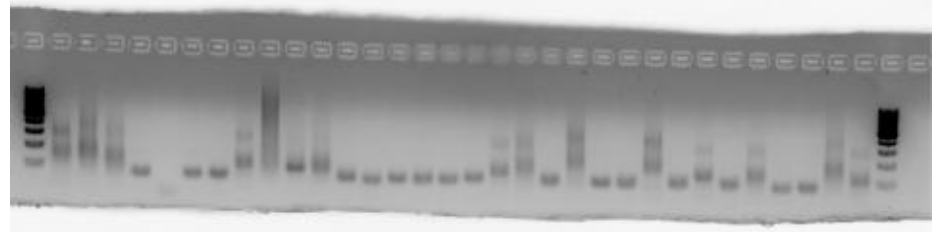
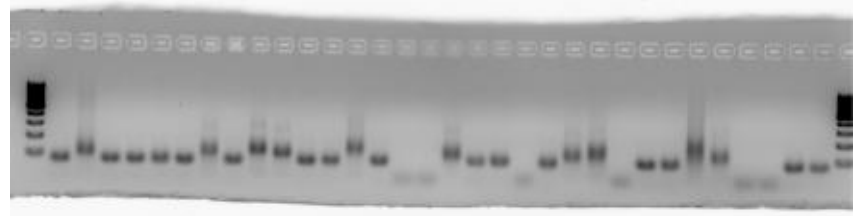
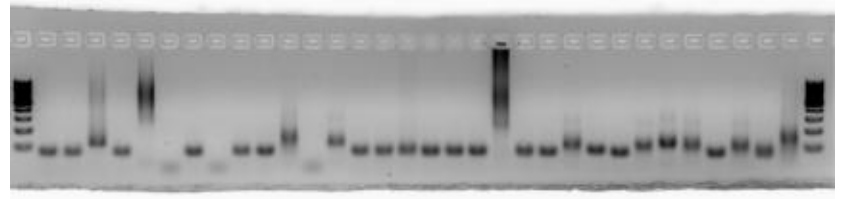
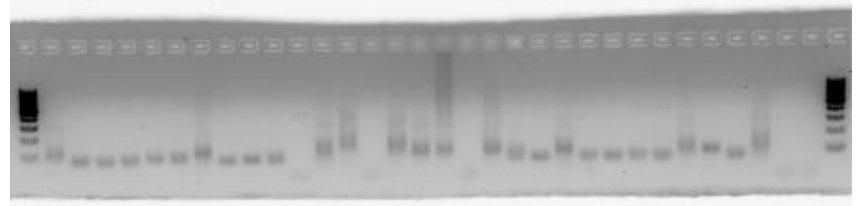
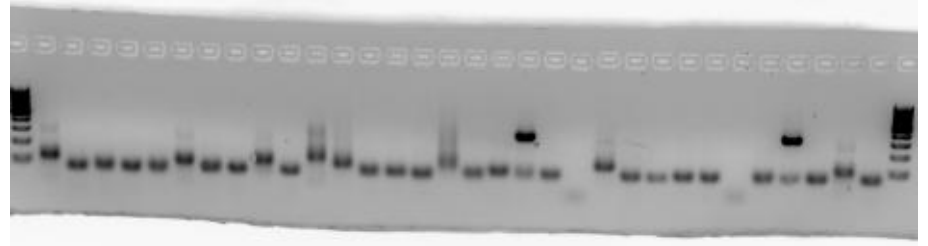
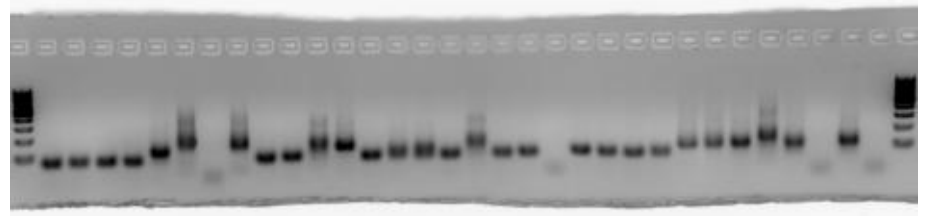
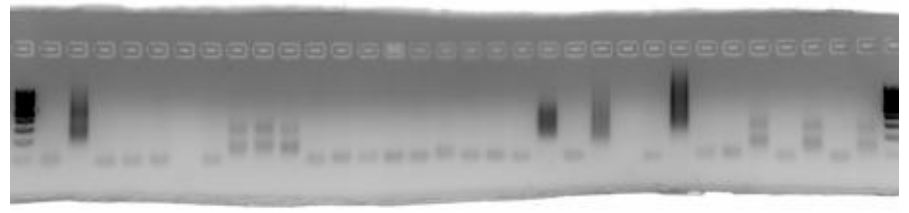

Large Young Oocytes

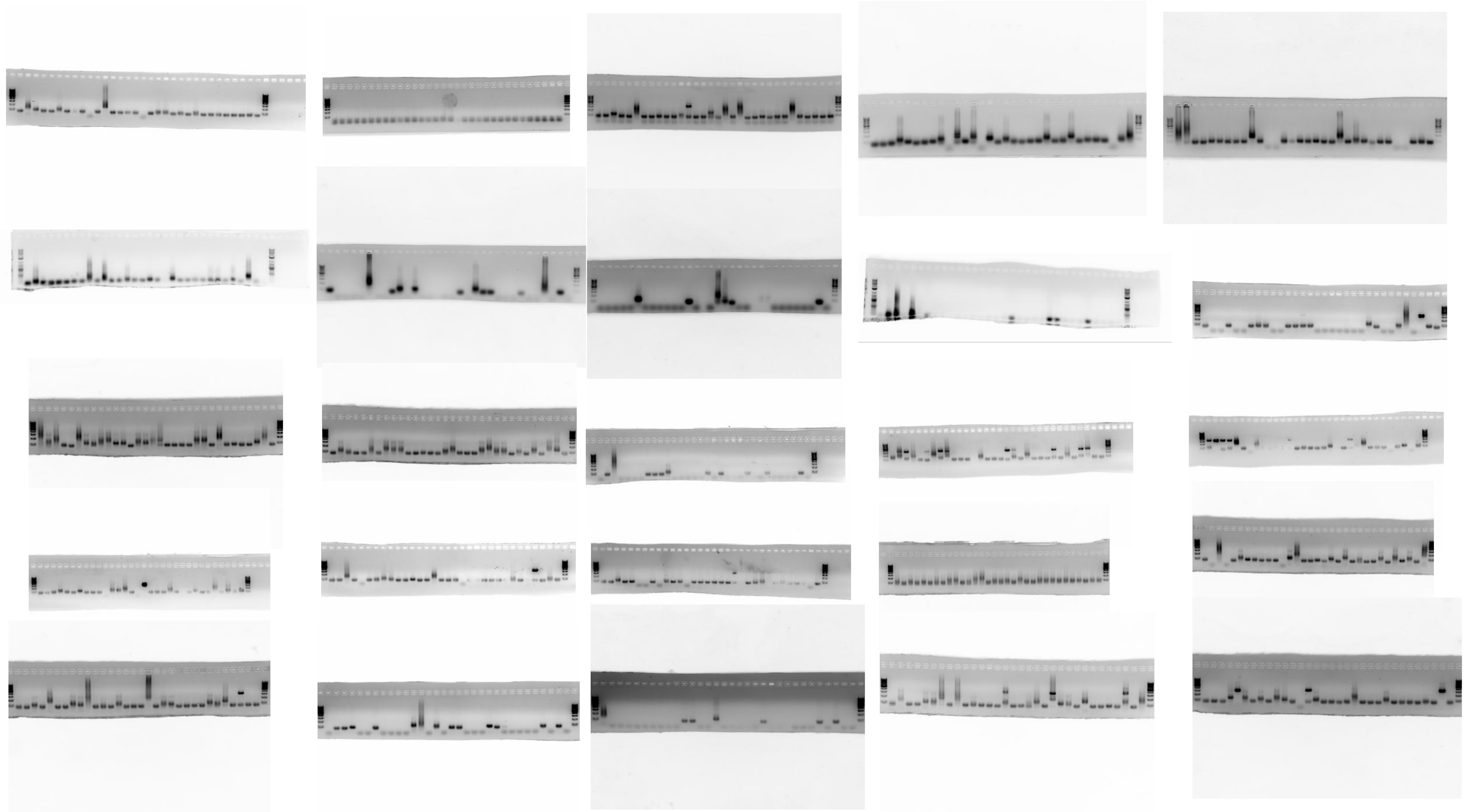

Large Young Oocytes

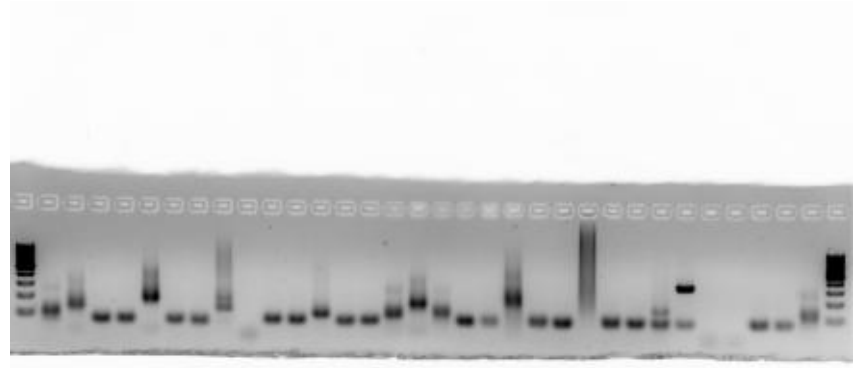
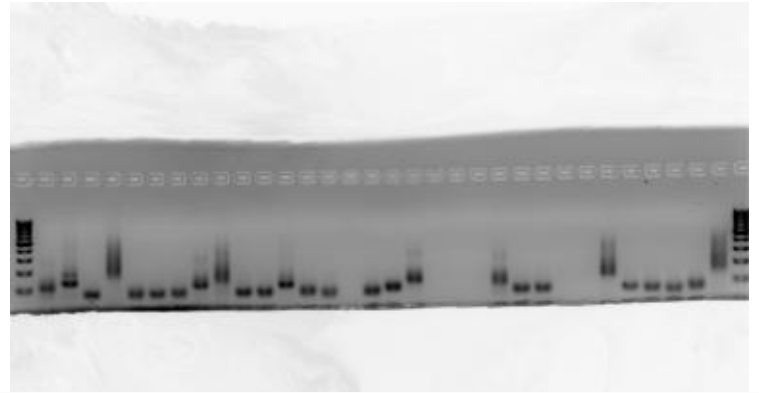
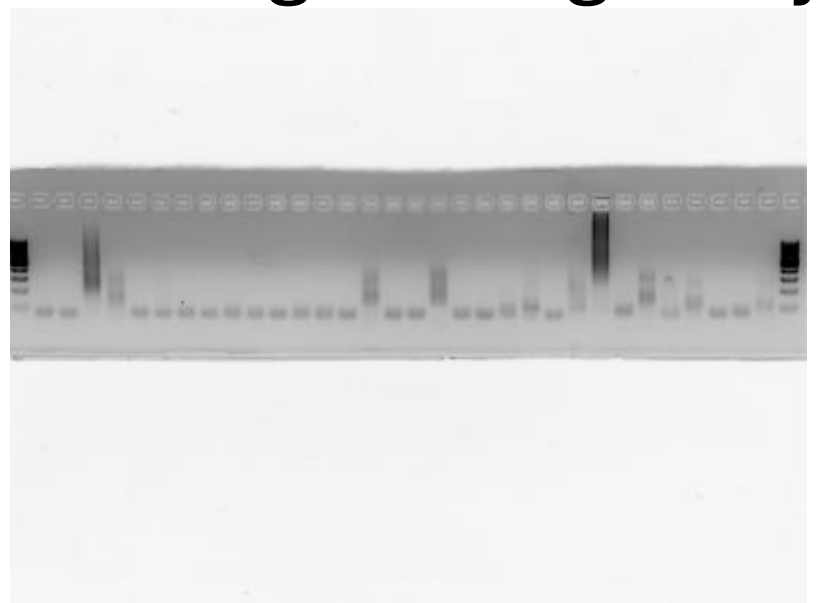
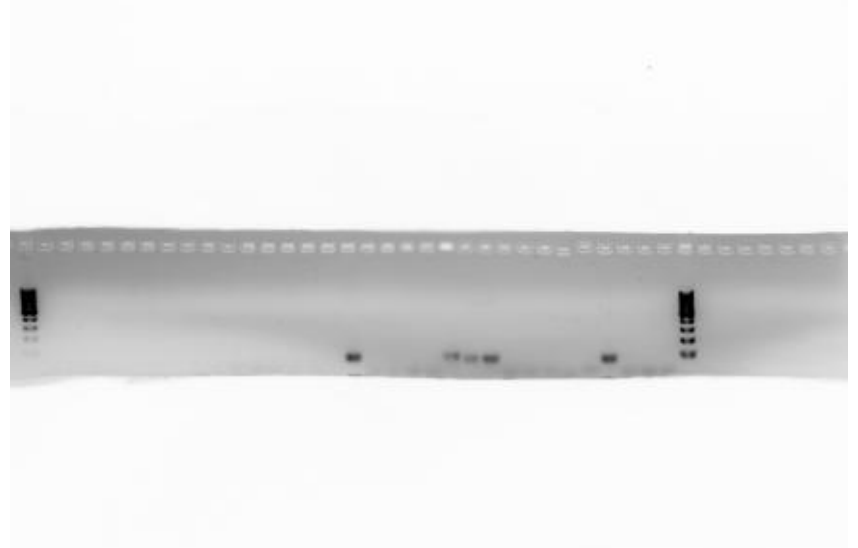
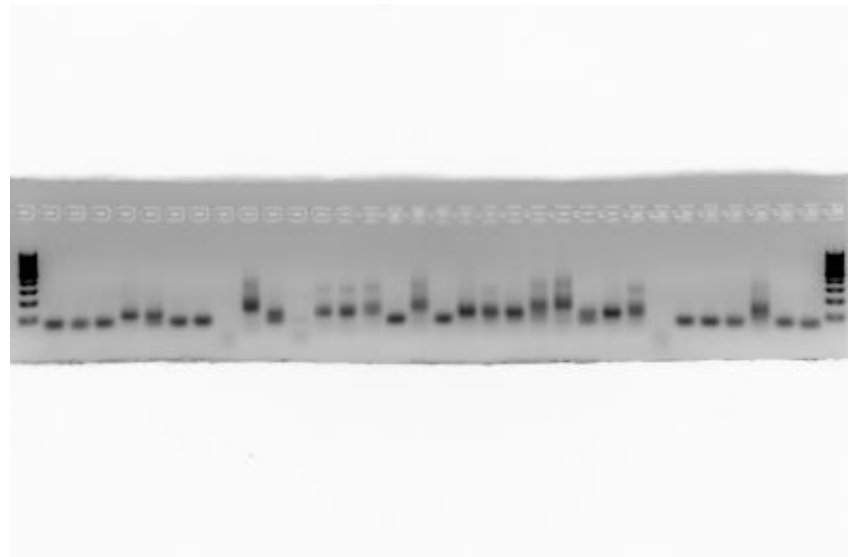
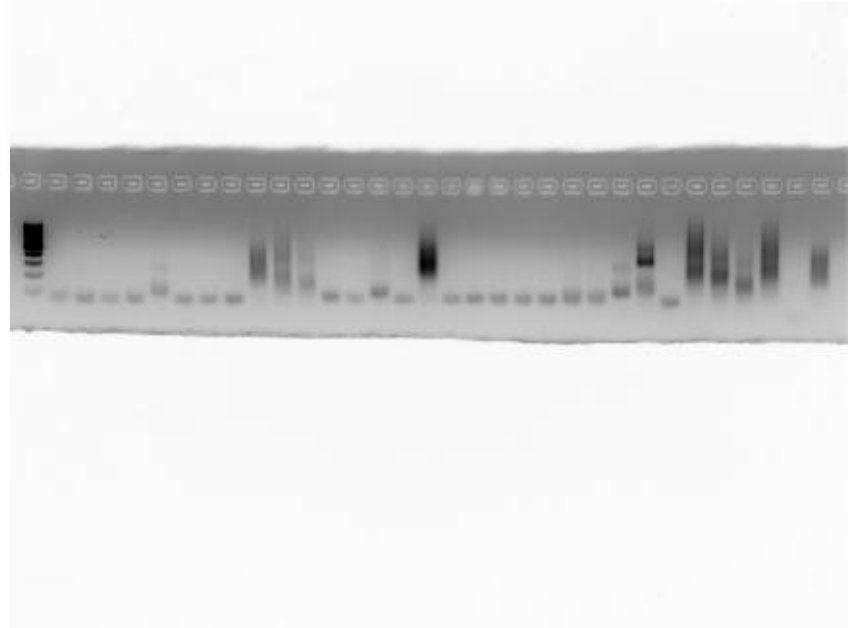
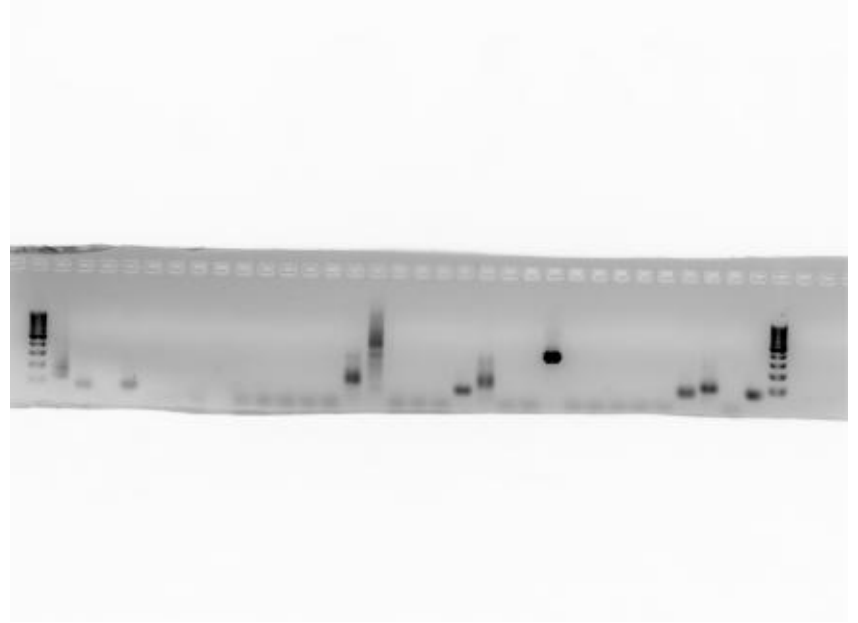
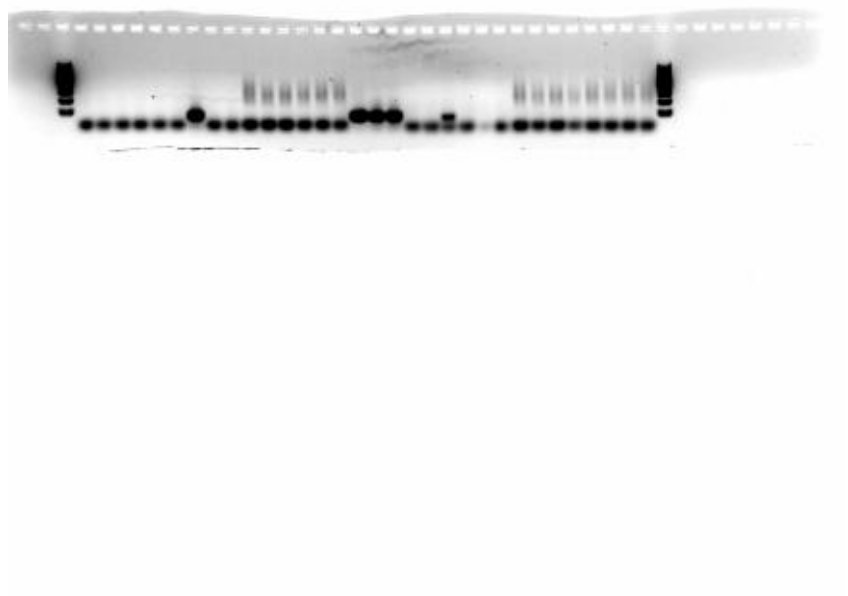
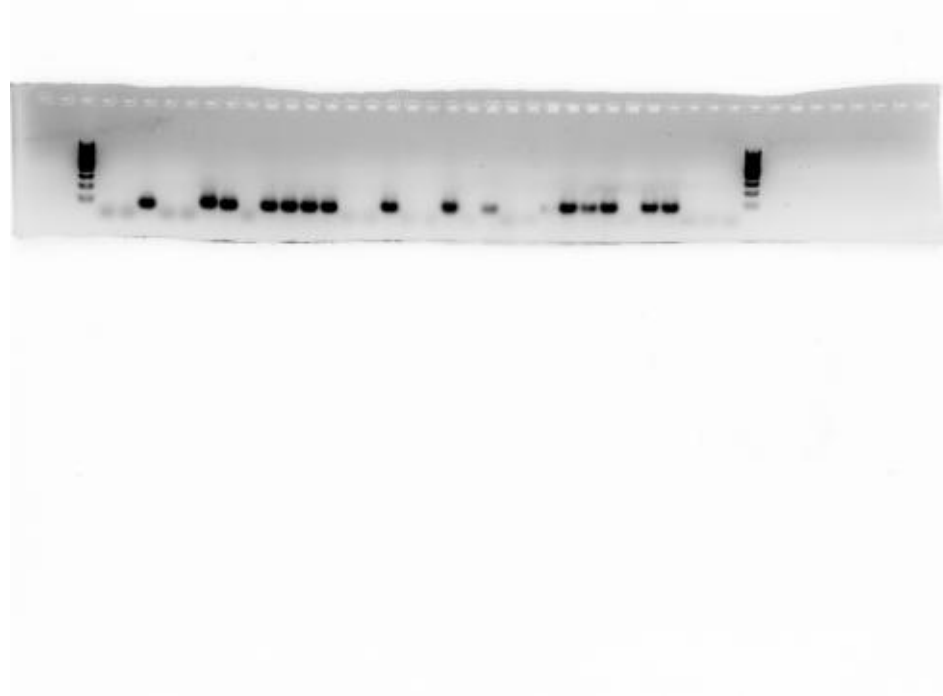
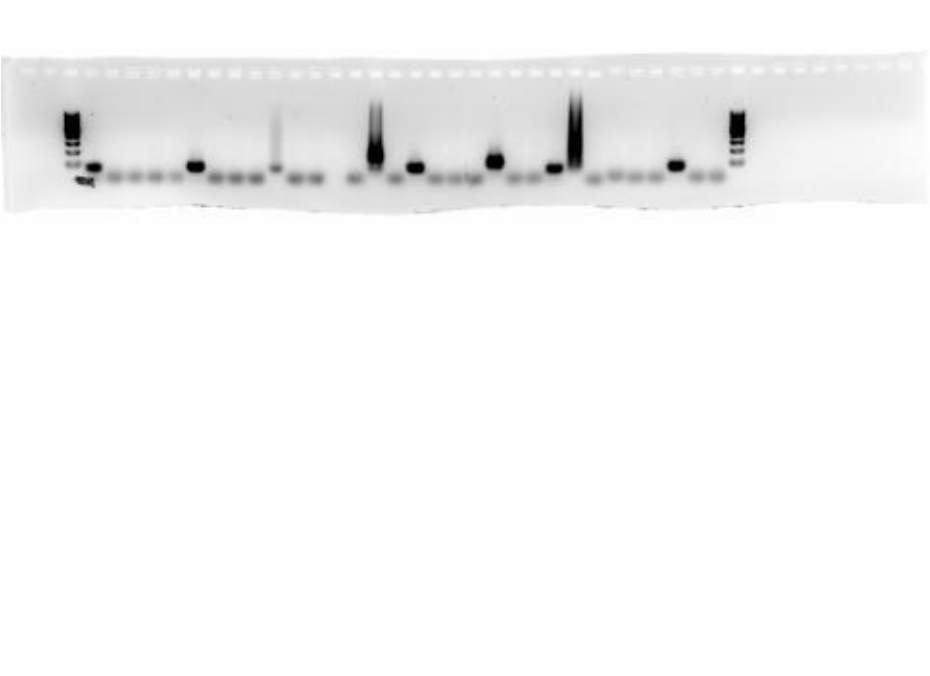

Low ΔΨm Young Oocytes

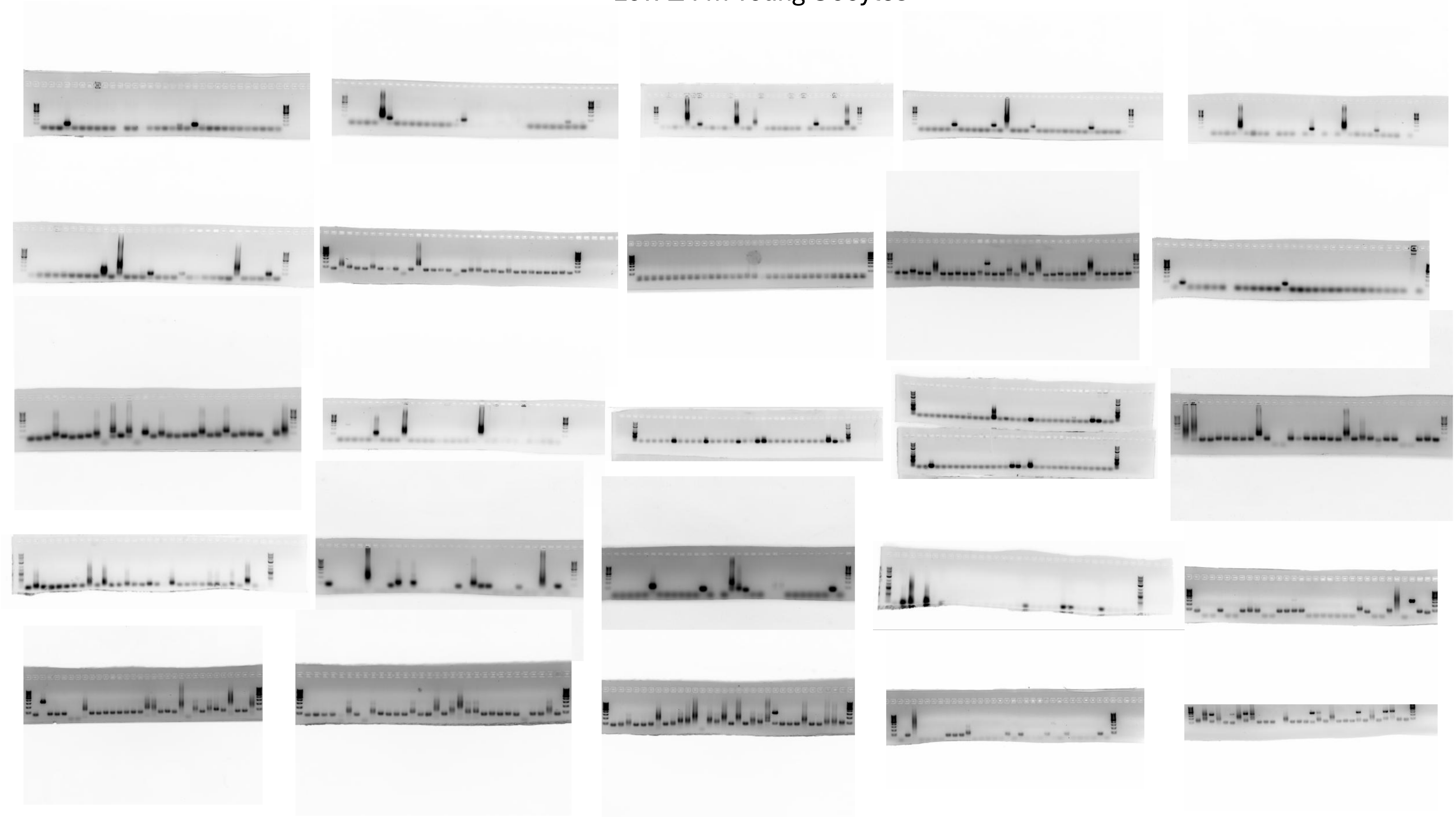

Low ΔΨm Young Oocyte

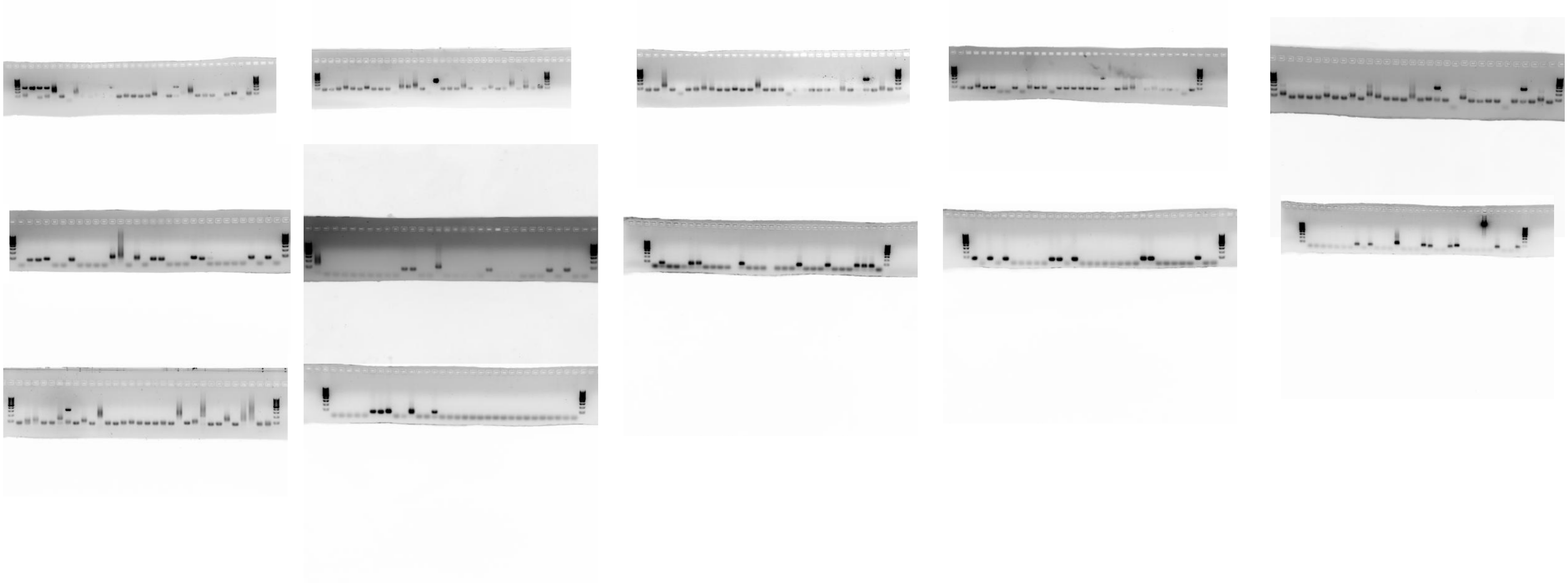

High ΔΨm Young Oocytes

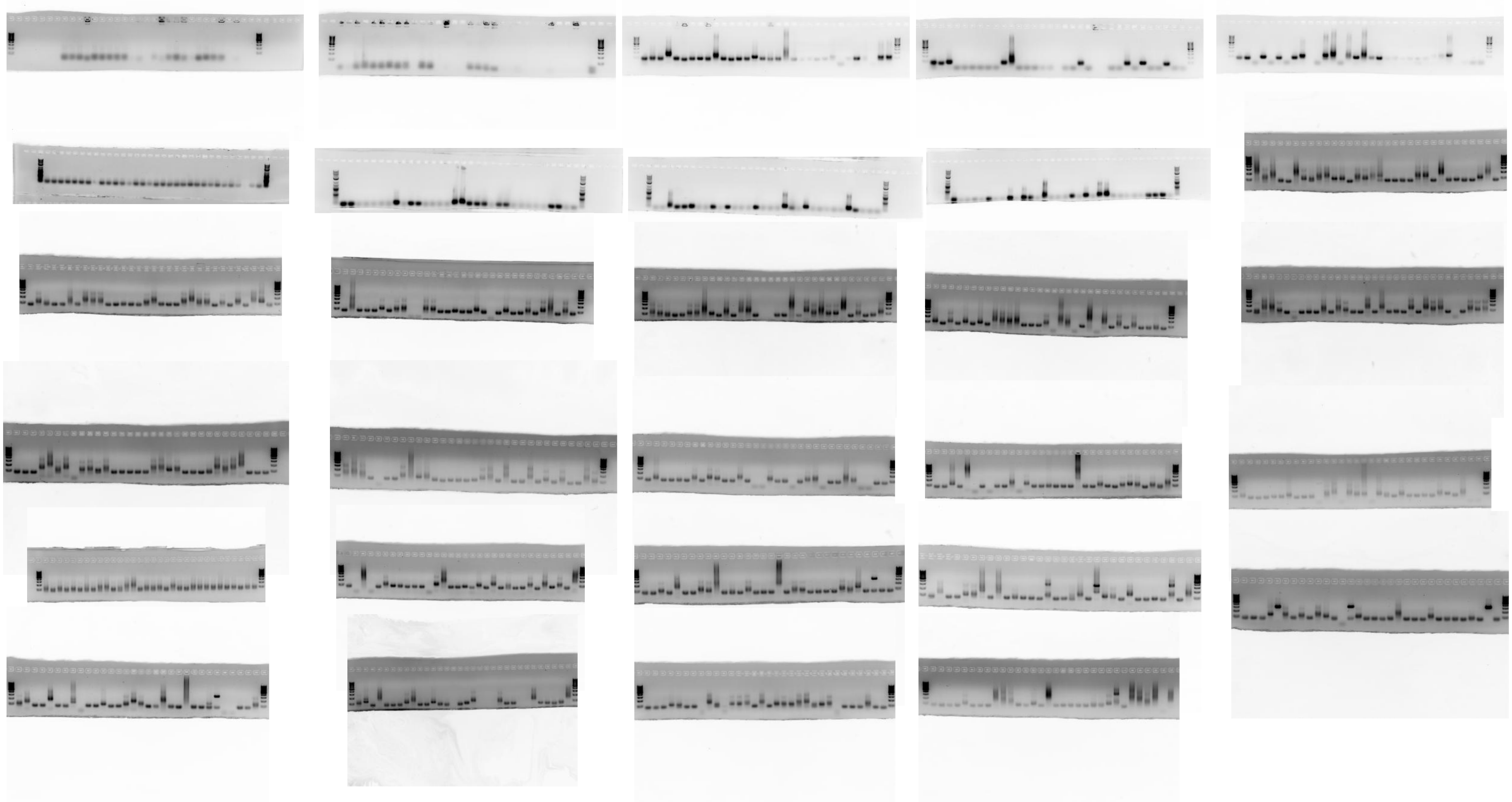

High ΔΨm Young Oocytes

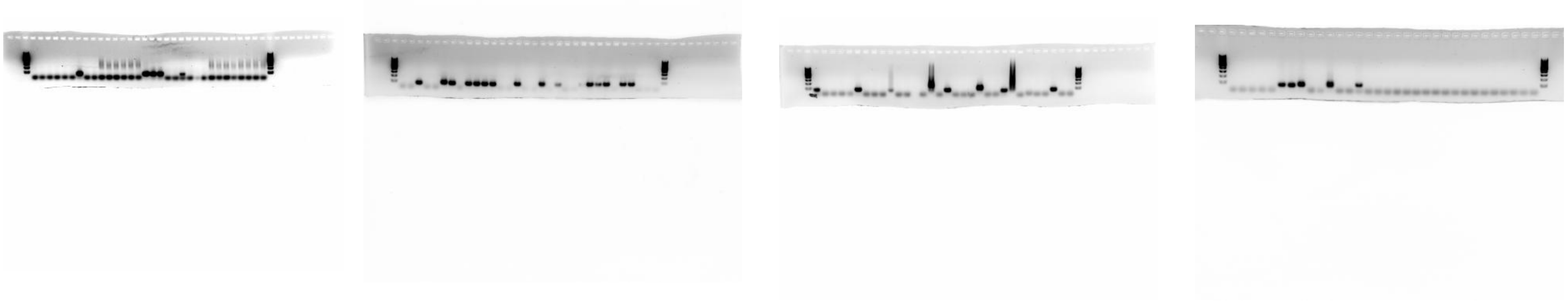

Supplement: Supplementary file 6 [file 13048_2026_2223_MOESM6_ESM.pdf]
