# Supplementary material for: Mitochondrial subpopulations in oocytes and cumulus cells exhibit distinct age-associated changes and selective plasticity in response to NMN supplementation
Source: J Ovarian Res. 2026 Aug 7;19:332. doi: 10.1186/s13048-026-02223-6 (PMC13732867; doi:10.1186/s13048-026-02223-6)
Supplement: Supplementary file 7 [file 13048_2026_2223_MOESM7_ESM.pdf]

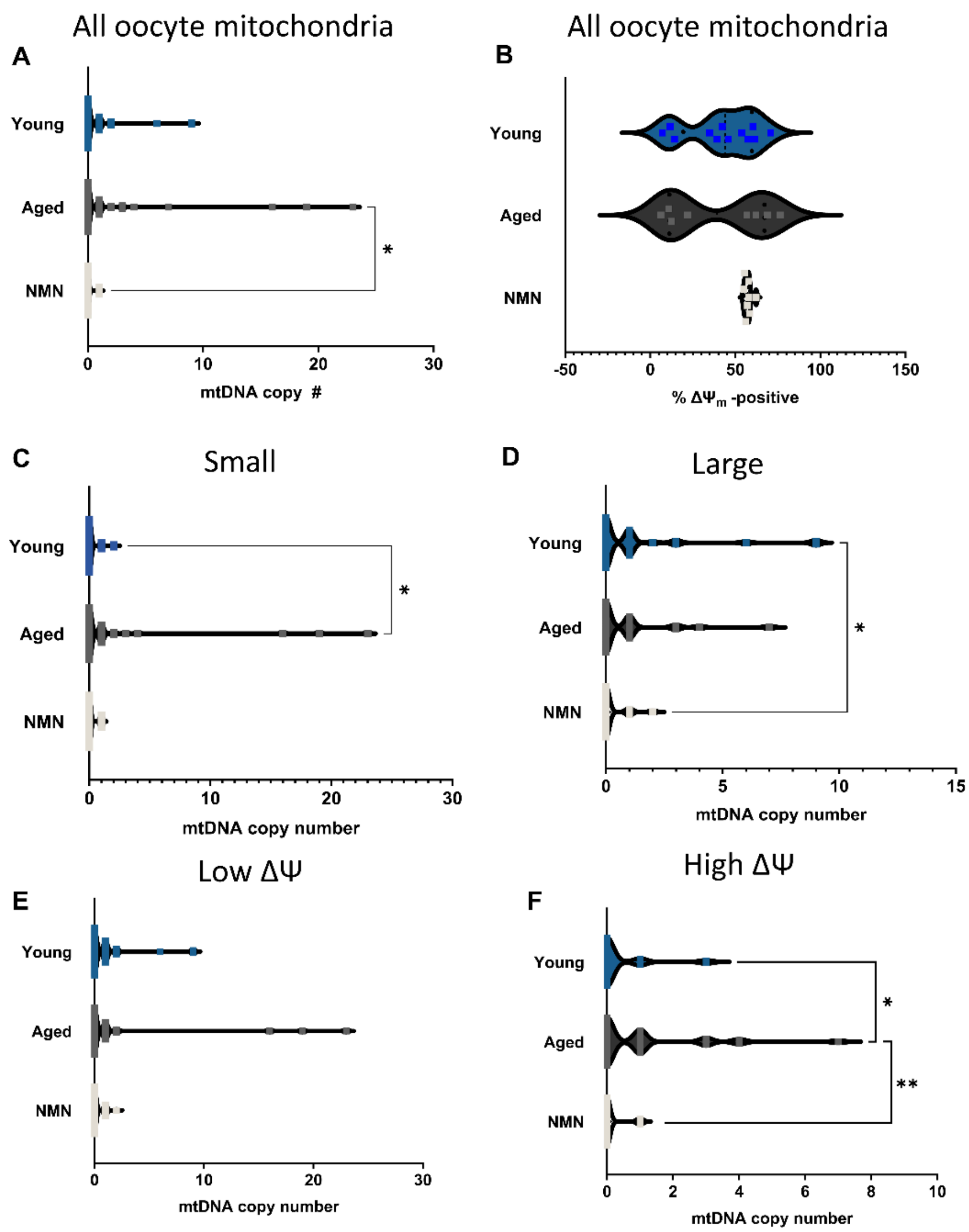

Supplemental Figure 1. Violin plots showing individual datapoints for oocyte mitochondria (A) mtDNA count for all oocyte mitochondria (mean = 0.732 copies per mitochondrion in young [n_{mice} = 6, $n_{mitochondria}$ = 56, SEM = 0.249], 1.484 copies per mitochondrion in aged [n_{mice} = 6, $n_{mitochondria}$ = 64, SEM = 0.527]) 0.147 copies per mitochondrion in NMN [n_{mice} = 6, $n_{mitochondria}$ = 34, SEM = 0.062], (B) percentage of ΔΨM positive mitochondria in oocytes (41.60% ΔΨM-positive in young [n_{mice} = 12, SEM = 6.092], 39.40% in aged [n_{mice} = 8, SEM = 10.360, $p > 0.999$], and 57.5% in NMN [n_{mice} = 8, SEM = 0.796]), (C) mtDNA count in small mitochondria (mean = 0.229 ± 0.092 for young, 1.756 ± 0.728 for aged, 0.208 ± 0.085 for NMN; [n_{mice} = 6, $n_{mitochondria}$ young], [n_{mice} = 6, $n_{mitochondria}$ = 45 aged; p = 0.0355], n_{mice} = 6, $n_{mitochondria}$ = 24 NMN p =

0.040 vs. aged untreated), (D) mtDNA count in large mitochondria (mean = 1.200 ± 0.389 for young, 0.788 ± 0.260 for aged, 0.208 ± 0.104 for NMN; [$n_{mice} = 6$, $n_{mitochondria} = 35$ young], [$n_{mice} = 6$, $n_{mitochondria} = 35$ aged; p = 0.5424], $n_{mice} = 6$, $n_{mitochondria} = 24$, NMN p = 0.017 vs. young), (E) mtDNA count in low ΔΨM mitochondria ([$n_{mice} = 6$, $n_{mitochondria} = 37$ young], [$n_{mice} = 6$, $n_{mitochondria} = 30$ aged], [$n_{mice} = 6$, $n_{mitochondria} = 24$ NMN-supplemented]), and (F) mtDNA count in high ΔΨM mitochondria(mean = 0.303 ± 0.134 for young, 1.000 ± 0.282 for aged, 0.125 ± 0.069 for NMN; [$n_{mice} = 6$, $n_{mitochondria} = 33$ young], [$n_{mice} = 6$, $n_{mitochondria} = 33$ aged; p = 0.0165 aged], [$n_{mice} = 6$, $n_{mitochondria} = 24$ NMN-supplemented; p = 0.0056 for aged vs. NMN-supplemented]). These data are the same as those shown in Figure 3.

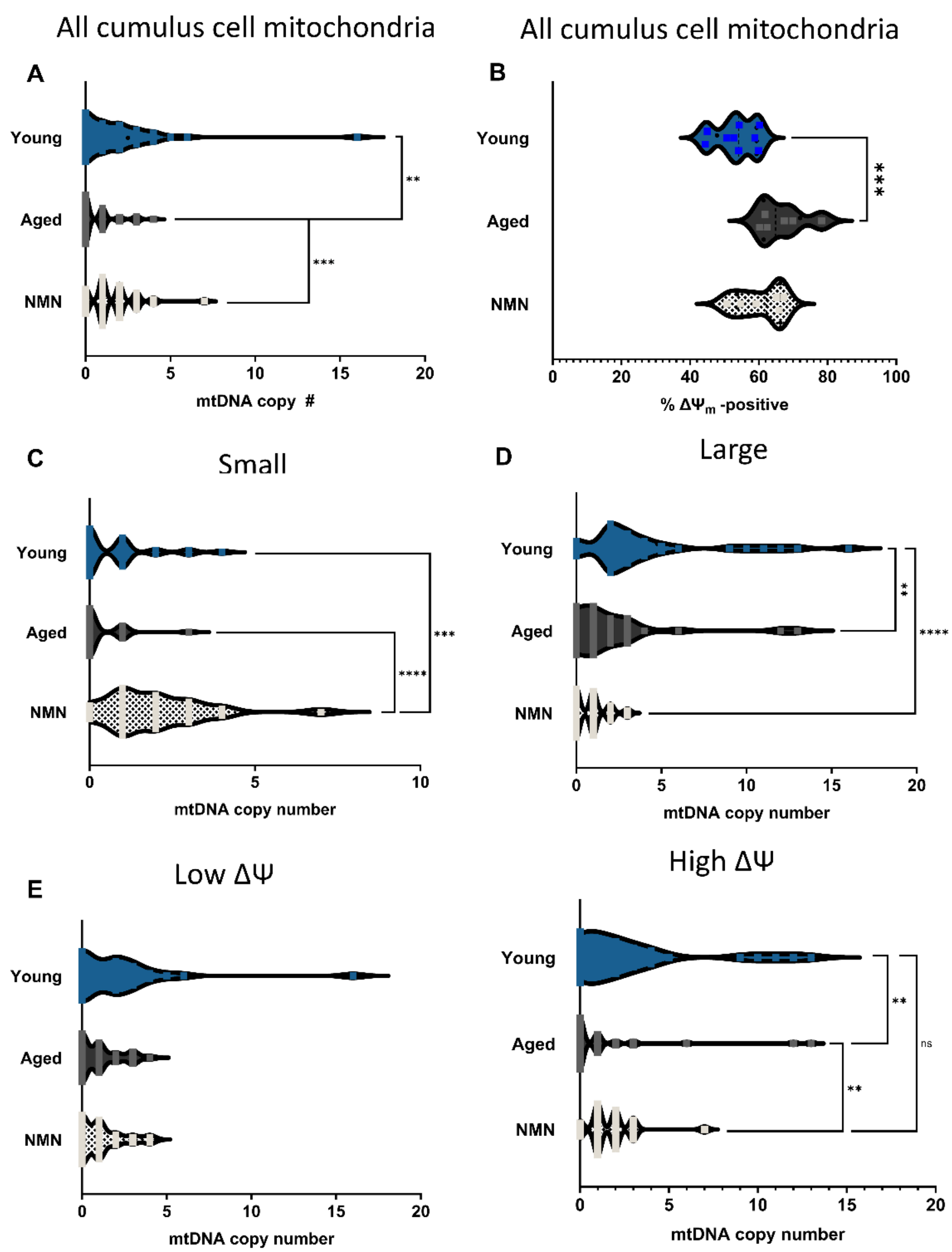

Supplemental Figure 2. Violin plots showing individual datapoints for cumulus cell mitochondria (A) mtDNA count for all cumulus cell mitochondria (mean = 1.736 copies per mitochondrion in young [n_{mice} = 6, $n_{mitochondria}$ = 53, SEM = 0.3441] vs. 0.7143 copies per mitochondrion in aged [n_{mice} = 6, $n_{mitochondria}$ = 63, SEM = 0.237]; p = 0.0054 vs. 1.649 copies per mitochondrion in NMN [n_{mice} = 6, $n_{mitochondria}$ = 37, SEM = 0.236]), (B) percentage of ΔΨM positive mitochondria in cumulus cells (55.33% $\Delta\Psi_M$-positive in young [n_{mice} = 9, SEM = 1.968] vs. 66.67% in aged [n_{mice} = 6, SEM = 2.769]; p = 0.0006) vs. 60.50% in NMN [n_{mice} = 6, SEM = 2.762], (C) mtDNA count in small mitochondria (mean = 0.774 ± 0.190 for young, 0.323 ± 0.117 for aged, 1.958 ± 0.321 for NMN; [n_{mice} = 6, $n_{mitochondria}$ = 31 young], [n_{mice} = 6, $n_{mitochondria}$ = 31 aged]; p = 0.0529, [n_{mice} = 6, $n_{mitochondria}$ = 24 NMN, p = 0.001 vs. young, p < 0.001 vs. aged]), (D) mtDNA count in large mitochondria (4.083 ± 0.648 for young, 2.156 ± 0.541 for aged, 0.8889 ± 0.180 for NMN; [n_{mice} = 6, $n_{mitochondria}$ = 36 young], [n_{mice} = 6, $n_{mitochondria}$ = 32 aged, p = 0.002], [n_{mice} = 6, $n_{mitochondria}$ = 27, NMN; p < 0.001 vs. young, p = 0.0703 vs. aged]) (E) mtDNA count in low ΔΨM mitochondria (mean = 2.125 ± 0.533 for young, 1.000 ± 0.213 for aged; [n_{mice} = 6, $n_{mitochondria}$ = 32 young], [n_{mice} = 6, $n_{mitochondria}$ = 31 aged]; p = 0.0844; mean = 1.07 ± 0.238 for NMN-supplemented, n_{mice} = 6, $n_{mitochondria}$ = 27), and (F) mtDNA count in high ΔΨM mitochondria (mean = 2.943 ± 0.616 for young, 1.500 ± 0.559 for aged, 1.750 ± 0.230 for NMN; [n_{mice} = 6, $n_{mitochondria}$ = 35 young], [n_{mice} = 6, $n_{mitochondria}$ = 32 aged; p = 0.0063], [n_{mice} = 6, $n_{mitochondria}$ = 24 NMN-supplemented; p = 0.0063 for aged vs. NMN-supplemented]). These data are the same as those shown in Figure 4.
